# Faecal microbiota transplant from aged donor mice affects spatial learning and memory via modulating hippocampal synaptic plasticity- and neurotransmission-related proteins in young recipients

**DOI:** 10.1186/s40168-020-00914-w

**Published:** 2020-10-01

**Authors:** Alfonsina D’Amato, Lorenzo Di Cesare Mannelli, Elena Lucarini, Angela L. Man, Gwenaelle Le Gall, Jacopo J. V. Branca, Carla Ghelardini, Amedeo Amedei, Eugenio Bertelli, Mari Regoli, Alessandra Pacini, Giulia Luciani, Pasquale Gallina, Annalisa Altera, Arjan Narbad, Massimo Gulisano, Lesley Hoyles, David Vauzour, Claudio Nicoletti

**Affiliations:** 1 Dept. of Pharmaceutical Sciences, University of Milan, Milan, Italy; 2 NEUROFARBA Department., University of Florence, Florence, Italy; 3 Earlham Institute, Norwich, UK; 4 Norwich Medical School, Biomedical Research Centre, University of East Anglia, Norwich, NR4 7TJ UK; 5 Dept. of Experimental and Clinical Medicine, University of Florence, 50134 Florence, Italy; 6 Dept. of Developmental and Molecular Medicine, University of Siena, Siena, Italy; 7 Neurosurgery Unit, Careggi University Hospital, Florence, Italy; 8 The Quadram Institute Bioscience, Norwich, UK; 9 Dept. of Biosciences, School of Science and Technology, Nottingham Trent University, Nottingham, UK

## Abstract

**Background:**

The gut-brain axis and the intestinal microbiota are emerging as key players in health and disease. Shifts in intestinal microbiota composition affect a variety of systems; however, evidence of their direct impact on cognitive functions is still lacking. We tested whether faecal microbiota transplant (FMT) from aged donor mice into young adult recipients altered the hippocampus, an area of the central nervous system (CNS) known to be affected by the ageing process and related functions.

**Results:**

Young adult mice were transplanted with the microbiota from either aged or age-matched donor mice. Following transplantation, characterization of the microbiotas and metabolomics profiles along with a battery of cognitive and behavioural tests were performed. Label-free quantitative proteomics was employed to monitor protein expression in the hippocampus of the recipients. We report that FMT from aged donors led to impaired spatial learning and memory in young adult recipients, whereas anxiety, explorative behaviour and locomotor activity remained unaffected. This was paralleled by altered expression of proteins involved in synaptic plasticity and neurotransmission in the hippocampus. Also, a strong reduction of bacteria associated with short-chain fatty acids (SCFAs) production (*Lachnospiraceae, Faecalibaculum*, and *Ruminococcaceae*) and disorders of the CNS (*Prevotellaceae* and *Ruminococcaceae*) was observed. Finally, the detrimental effect of FMT from aged donors on the CNS was confirmed by the observation that microglia cells of the hippocampus fimbria, acquired an ageing-like phenotype; on the contrary, gut permeability and levels of systemic and local (hippocampus) cytokines were not affected.

**Conclusion:**

These results demonstrate that age-associated shifts of the microbiota have an impact on protein expression and key functions of the CNS. Furthermore, these results highlight the paramount importance of the gut-brain axis in ageing and provide a strong rationale to devise therapies aiming to restore a young-like microbiota to improve cognitive functions and the declining quality of life in the elderly.

Video Abstract

## Background

Ageing leads to the loss of functional capacity in several body systems, including the cardiovascular, skeletomuscular, osteoarticular and neuro-immune-endocrine, and is often associated with a decline in psychological wellbeing and cognitive function. In the past few years, it has been brought to the surface that events taking place in the gut play an important role in the ageing process [1], and recently, the existence of bidirectional communication between the gut and the brain—the gut-brain axis—has emerged as an important player in shaping aspects of behaviour and cognitive function [2]. In particular, the gut microbiome has been reported to play an important role within this scenario. A modest alteration in the composition of the gut microbiota, induced by either diet or antibiotics, is sufficient to cause changes in mouse brain chemistry and function [3, 4]. In particular, the oral administration of antibiotics resulted in an increase in brain-derived neurotrophic factor (BDNF) in the hippocampus. Furthermore, reduced expression of the synaptic plasticity-related genes PSD-95 and synaptophysin, important in memory formation and maintenance, were also reported [5]. Yet, germ-free (GF) mice are less responsive to exposure of environmental stressors compared to conventionally reared mice, with conventionalization of GF animals impacting significantly on brain development [6]. This observation, along with a growing body of evidence, has shown that the gut microbiota plays a major role in in the development and function of the CNS, affecting learning and memory via metabolic, neuroendocrine and immune pathways [7]. In addition, dysbiosis has been associated with a variety of neurological disturbances ranging from depression [8] to autism [9, 10], along with neurodegenerative diseases such as Parkinson’s disease and multiple sclerosis [11, 12]. Not surprisingly, faecal microbiota transplant (FMT) is being investigated as a therapeutic option not only for GI-tract-related diseases [13], but also for CNS disorders [14, 15]. Thus, it is plausible to hypothesize that age-associated changes in the microbiota composition have a direct effect on the CNS, potentially contributing to the decline of cognitive function seen in the elderly. Recently, it has been reported that FMT from aged donor mice into young germ-free (GF) recipients increased hippocampal neurogenesis, intestinal growth and activation of the prolongevity signalling pathways in the liver [16]; however, up to date, the direct impact of the ageing microbiota on cognitive functioning and activity has not been addressed. We report that FMT from aged mice led to a decline of spatial learning and memory in young adult (henceforth termed adult) recipients via modulation of synaptic plasticity- and neurotransmission-related proteins in the hippocampus, an area of the CNS known to be heavily affected by the ageing process [17]. The impact of age-associated shifts of microbiota on the CNS was further confirmed by the observation that microglia cells of the hippocampus fimbria of adult mice acquired an ageing-like phenotype after FMT from aged donors. These changes occurred in the absence of an overt increase of both plasma and hippocampal levels of inflammatory cytokines and intestinal permeability.

## Results

### Microbiota of adult recipients acquires an aged phenotype following FMT from aged donors

**Fig. 1:**
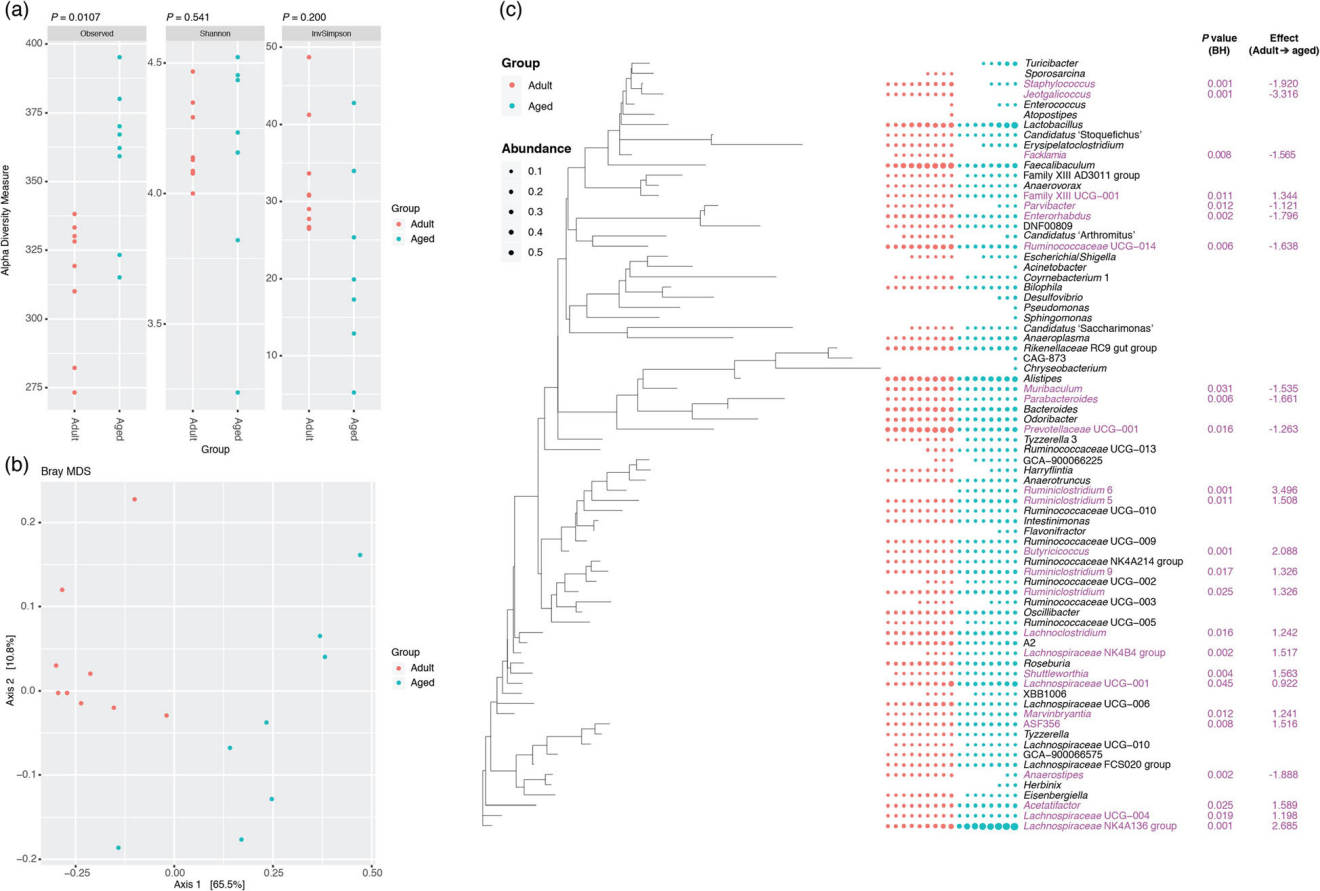
Comparison of the faecal microbiotas of adult and aged mice. **a** Measures of alpha diversity. Significance of differences between the two groups was assessed by Wilcoxon rank sum test. **b** MDS plot of a Bray-Curtis assessment of beta diversity. Data presented are for ASVs present in more than two animals and prevalence threshold of 1% at the genus level. **c** Comparison of the relative abundance of different genera present in the faecal microbiota of the two cohorts. Purple text, significantly different (Welch’s *t* test and Wilcoxon; *P* < 0.05, Benjamini-Hochberg) based on ALDEx2 analyses

**Fig. 2:**
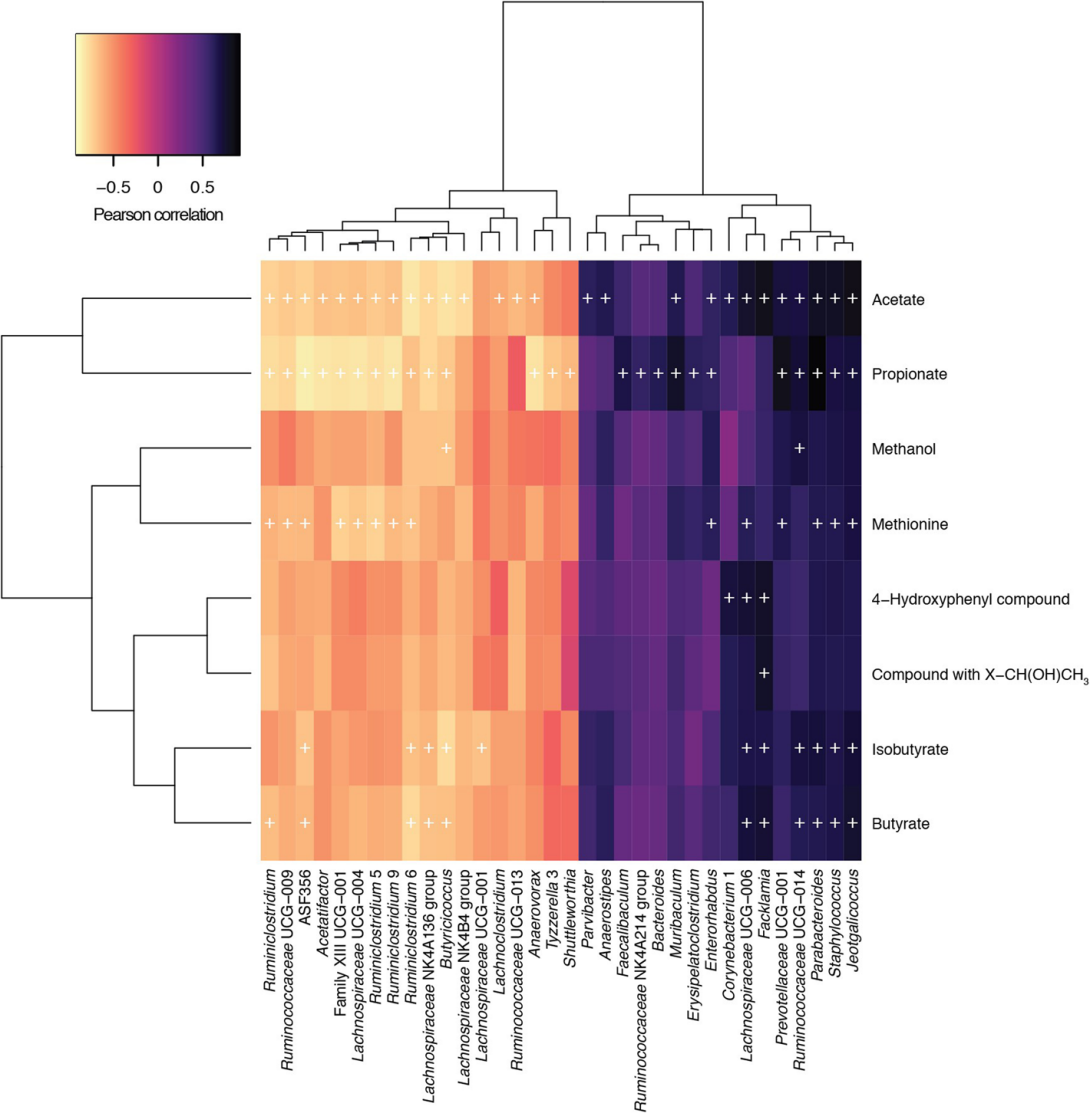
Pearson correlation of faecal microbiomic and metabolomic data for adult and aged mice. ALDEx2 was used to correlate the datasets. +, *P* < 0.05 (Benjamini-Hochberg). Only rows/columns containing significant data are shown. (8 mice/group)

**Fig. 3:**
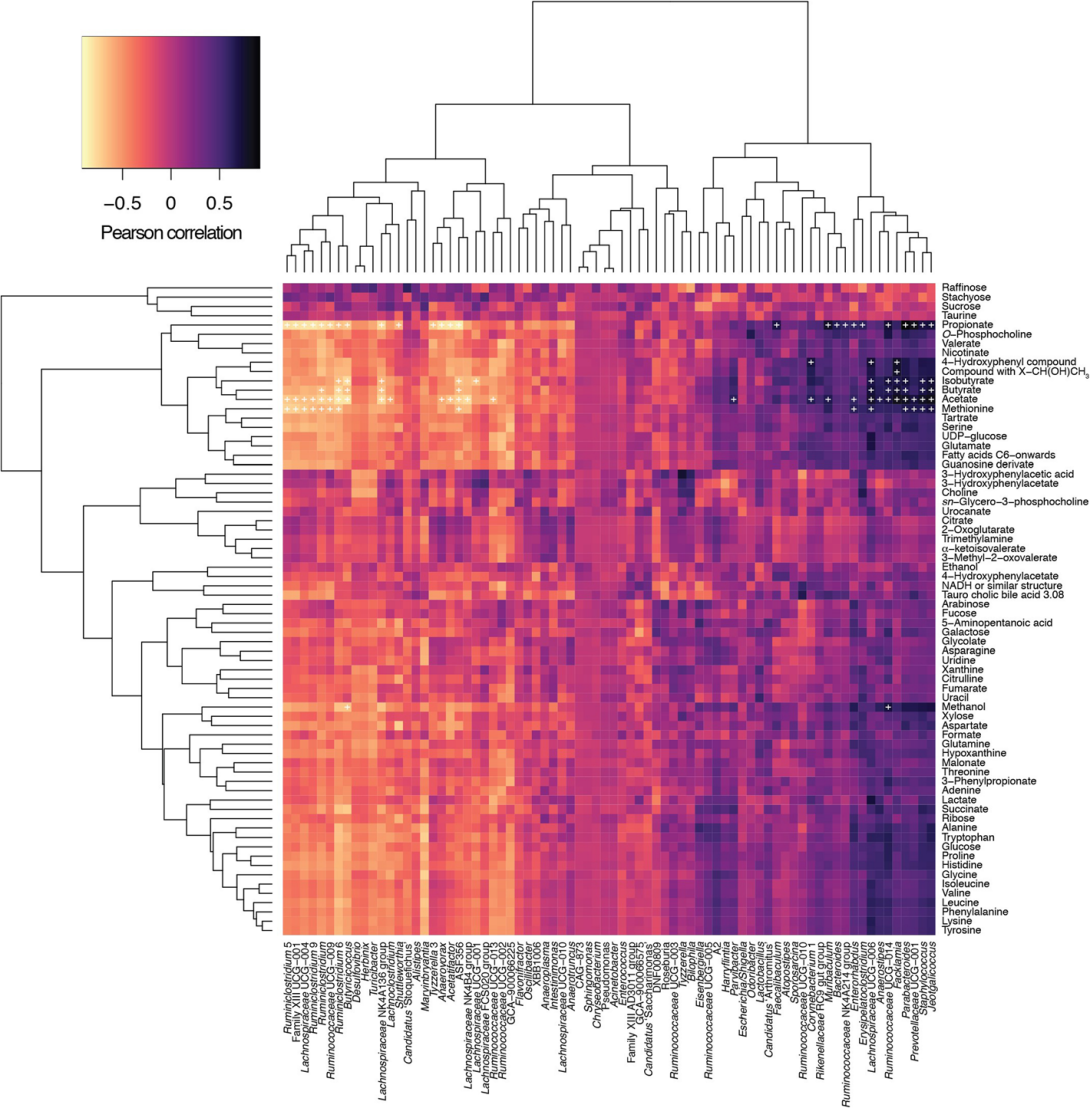
Pearson correlation of faecal microbiomic and metabolomic data for adult and aged mice. ALDEx2 was used to correlate the datasets. +, *P* < 0.05 (Benjamini-Hochberg). All data are shown (8 mice/group)

**Fig. 4:**
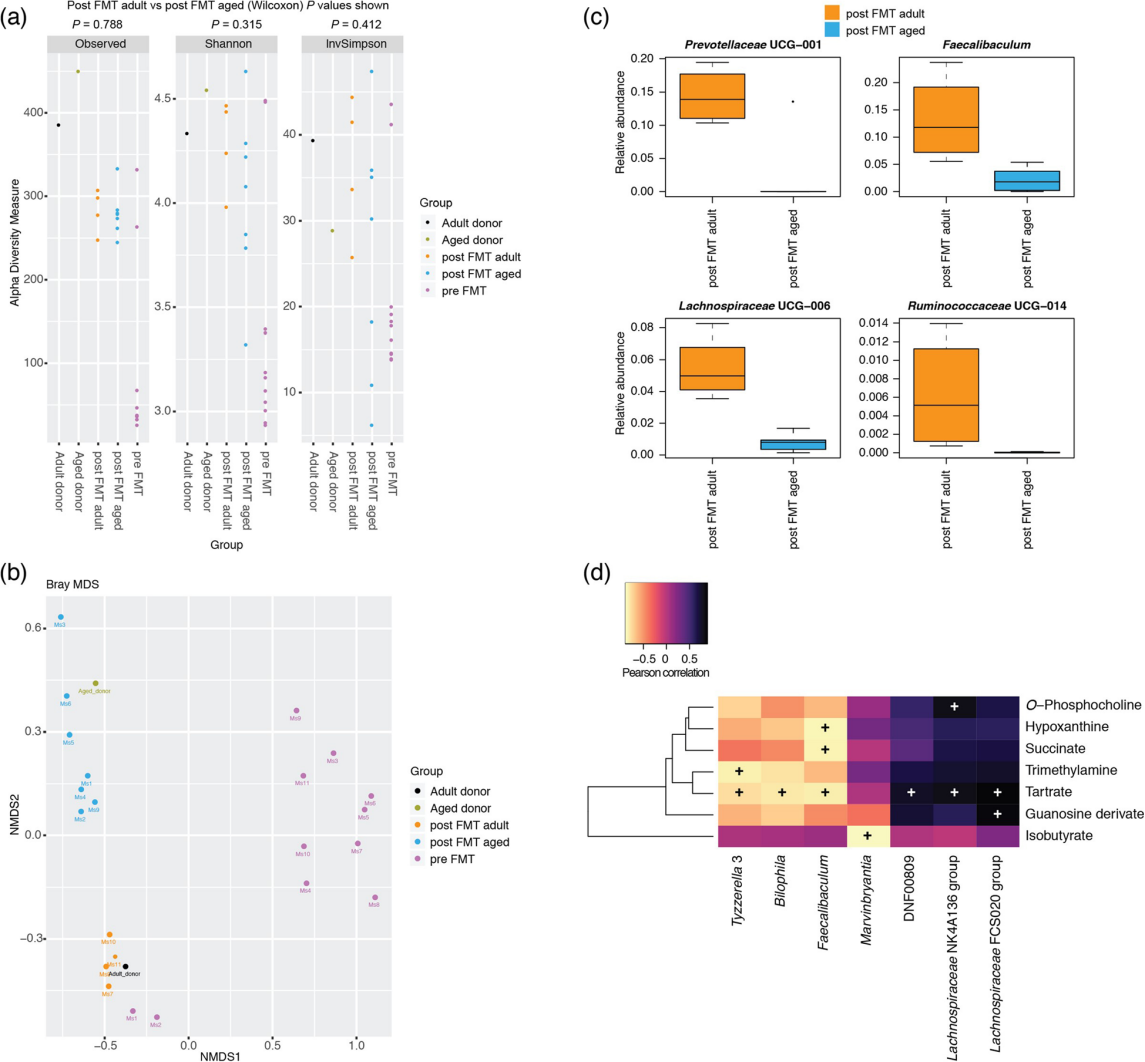
Effect of FMT on the faecal microbiotas of adult mice. **a** Measures of alpha diversity among the pooled adult (*n* = 1) and aged (*n* = 1) samples, the adult mice pre FMT (*n* = 11) and the adult mice after FMT with adult (*n* = 4) and aged (*n* = 7) faeces. **b** MDS plot of a Bray-Curtis assessment of beta diversity. Data presented are for ASVs present in more than two animals and prevalence threshold of 1% at the genus level. **c** Box plots for the genera that were significantly different (Welch’s *t* test and Wilcoxon; *P* < 0.1, Benjamini-Hochberg) between post FMT adult (*n* = 4) and post FMT aged (*n* = 7) mice based on ALDEx2 analyses. **d** Pearson correlation of faecal microbiomic and metabolomic data. Only rows/columns that contained significant data (*P* < 0.1, Benjamini-Hochberg) are shown

We investigated in the first instance the faecal microbiotas of adult (3 months) and aged mice (24 months) (Fig. 1). Measures of alpha diversity showed only a significant (*P* = 0.0107) difference in species richness between the two cohorts, with the faecal microbiota of aged mice harbouring more different amplicon sequence variants (ASVs) than adult mice (Fig. 1a). Beta diversity (Bray-Curtis) analysis showed clear separation of the two groups of mice based on their faecal microbiotas (Fig. 1b). Comparison of relative abundances of different taxa showed significantly more ASVs associated with *Ruminiclostridium*, *Butyricicoccus*, *Lachnoclostridium*, *Lachnospiraceae* spp., *Shuttleworthia* and *Marvinbryantia*, and significantly fewer associated with *Staphylococcus*, *Jeotgalicoccus*, *Facklamia*, *Parvibacter*, *Enterorhabdus*, *Muribaculum, Parabacteroides* and *Anaerostipes* in the adult mice relative to the aged mice (Fig. 1c). Integration of faecal microbiomic and metabolomic data showed an association between lower levels of faecal short-chain fatty acids (SCFAs) (Fig. 2) and decreased representation of obligate anaerobes such as the *Lachnospiraceae* and *Ruminococcaceae* (Fig. 3). The above approach was paralleled by the metabolomics analysis of the intestinal luminal contents. Using 1H-NMR to profile luminal metabolites, we were able to obtain insights into microbiota metabolism and function that can only be inferred from metagenomics or transcriptomics approaches. Using Principal Component Analysis to compare and contrast 89 metabolites (including amino acids, organic acids, sugars, nucleosides and short-chain fatty acids), clear separations of the metabolic profile detected in conventionalized adult and aged mice were apparent (PC1) (Additional file 1). Based on this analysis, faeces from three of the adults and two of the aged mice representative of the two groups were pooled and used as donors for FMT (Additional file 1). To this end, it is important to stress that many studies have employed germ-free animals to determine the role of the gut microbiota in modulating aspects of host physiology, but this approach is however not suitable for our purposes. Germ-free mice bear significant abnormalities in BBB structure and integrity [18] and in microglial numbers and morphology [19] profoundly complicating interpretation of the effects of FMT in our model. We have therefore chosen to use antibiotic-mediated microbial depletion to examine the mediating role of the gut microbiota, as while removal of gut microbes may not be as comprehensive as in germ-free animals, we have avoided the confounding effects caused by development in an entirely microbe-free environment. Adult mice received FMT either from adult donors (FMT-adult) or aged donors (FMT-aged). Groups of transplanted mice were then used to assess the post-FMT microbiota profile. Measures of alpha diversity showed no significant differences between the two mouse sub-groups (Fig. 4a). Bray-Curtis analysis showed clear separation of the pre- and post-FMT groups, with the post-FMT samples clustering with their respective inoculants (Fig. 4b). It is notable that antibiotic treatment of pre-FMT mouse 1 (Ms1) and mouse 2 (Ms2) was not completely successful as they clustered more closely with the post-FMT adult group and adult pooled sample. However, after these mice had been inoculated with the adult pooled sample, they clustered with the aged post-FMT group. The differences in the faecal microbiotas between the adult and aged mice post-FMT were not as pronounced as the differences seen between the adult and aged groups in our initial study; as such a less stringent Benjamini-Hochberg cut-off (*P* < 0.1) was used when analysing these data (Fig. 1). This less-pronounced change is unsurprising as the adult and aged mice in the initial study had not been subject to any intervention; however, it is becoming clear that even subtle changes in the gut microbiome are associated with phenotypic changes in the host [20] and the same may be true of cognitive traits. Only four genera (*Prevotellaceae*, *Faecalibaculum*, *Lachnospiraceae* and *Ruminococcaceae*) were found to be significantly differentially abundant in the faeces of the post-FMT adult and post-FMT aged animals (Fig. 4c). Few significant associations were seen between the faecal microbiomic and metabolomic data (Fig. 4d).

### FMT from aged mice to adult recipients results in decreased spatial learning and memory but does not affect anxiety-like behaviour or motor activity

**Fig. 5:**
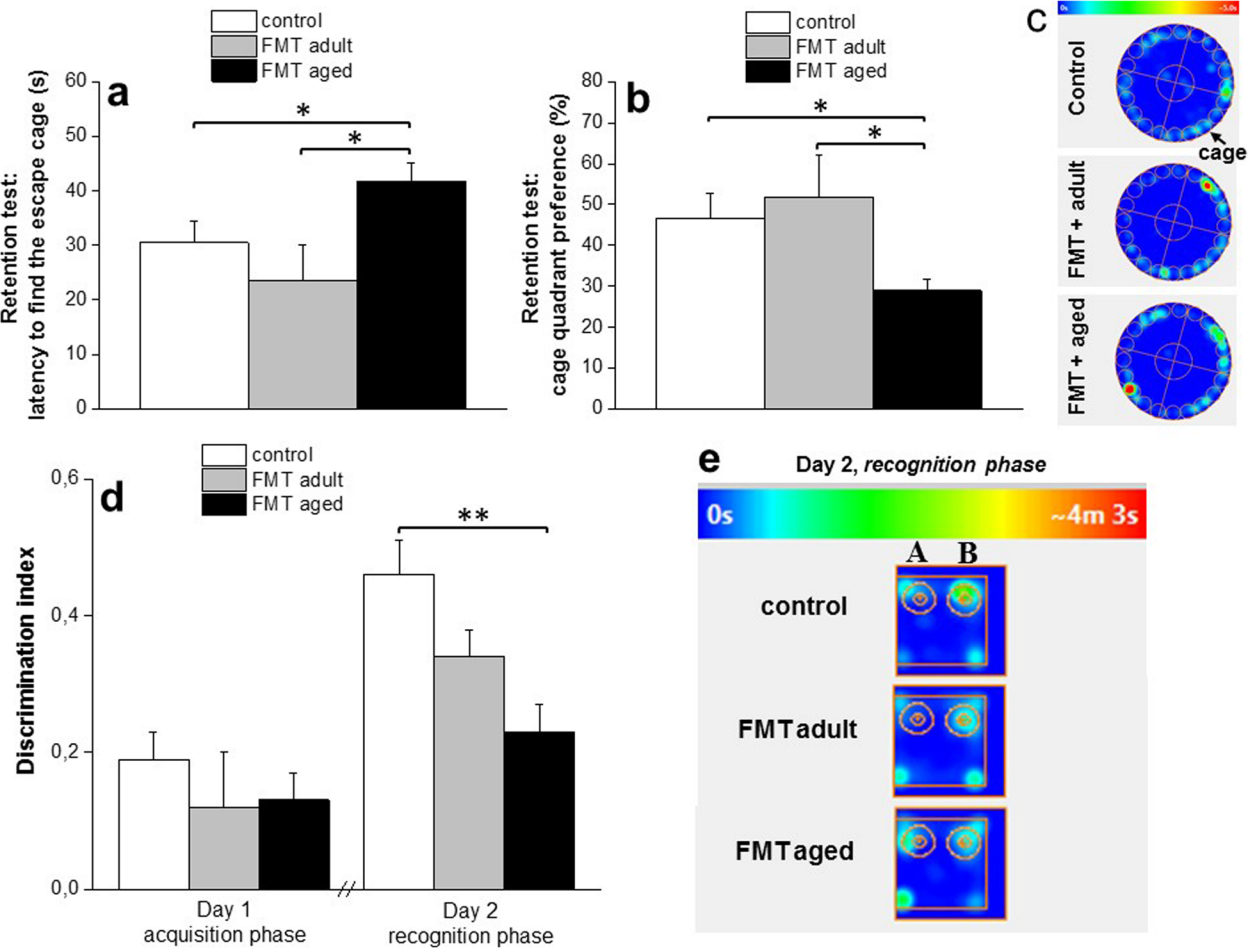
Effect of FMT from adult and old mice on spatial learning and memory. Barnes maze test (Fig. 3a–c): mice were trained to find the cage for 4 consecutive days (twice daily; 2 trials). The average primary latency (Fig. 3a) was significantly higher for adult recipients of microbiota from aged donors (FMT-aged) than the other control groups either left untreated or colonized with microbiota from adult, age-matched donors (FMT-adult). Furthermore, during the retention test, FMT-aged mice spent less time in the target quadrant that contained the escape tunnel compared to control groups (Fig. 3b; heat map in Fig. 3c). The values represent the mean ± SEM for each group (*n* = 10–12 mice/group). *P < 0.05 vs control animals and FMT-adult. The novel object recognition test (Fig. 3d–e): on day 1, mice were exposed to two similar objects (A + A); on day 2, animals were re-exposed to the testing area containing one novel object (A + B). The time spent by the animals exploring each object was recorded. The discrimination index, calculated as (TB-TA)/(TB + TA), was used to assess the preference for the novel object. Control groups, either untreated or FMT-adult mice, preferred the novel object more than the familiar one, whereas FMT-aged mice showed a significant reduction in the time spent exploring the novel object (heat map in 3e) suggesting reduced discrimination as a consequence of impaired memory capabilities. The values represent the mean ± SEM for each group (*n* = 10–12 mice/group). **P < 0.01 vs control animals

In the first set of experiments, we investigated the impact of FMT from aged mice to adult recipients to a series of spatial learning and memory tests. First, the Barnes maze test was used to assess memory and spatial learning. Following the training trials, a retention test was conducted in which the escape tunnel was removed and the latency before moving to the position of the former escape tunnel for the first time was measured. We observed that the average primary latency was significantly higher for FMT-adult recipients of microbiota from aged donors (FMT-aged) than the other control groups either left untreated or colonized with microbiota from adult, age-matched donors (FMT-adult) (41.7 ± 3.5 s; 30.5 ± 3.9 s and 23.5 ± 6.7 s, respectively; *P* < 0.05) (Fig. 5a). Yet, during the retention test, FMT-aged mice spent less time in the target quadrant (28.9 ± 2.6 s in comparison to control 46.5 ± 6.3 s, and FMT-adult 51.8 ± 10.2 s; *P* < 0.05) that contained the escape tunnel compared to control group (Fig. 5b; heat map in Fig. 5c) confirming an important role of the gut microbiota on memory and spatial learning. Learning and memory was further evaluated by the novel object recognition test. Significant differences between groups were observed in the time spent exploring two different objects (Fig. 5d). In particular, the time spent exploring novel and familiar objects was monitored; the object recognition was assessed by a defined discrimination index. Control groups, either untreated or FMT-adult mice, preferred the novel object more than the familiar one, whereas FMT-aged mice showed a significant reduction in the time spent exploring the novel object compared to control (0.23 ± 0.04 and 0.46 ± 0.05, respectively; *P* < 0.01) suggesting reduced discrimination as a consequence of impaired memory capabilities. This is further evidenced by the heat maps (Fig. 5e).

**Fig. 6:**
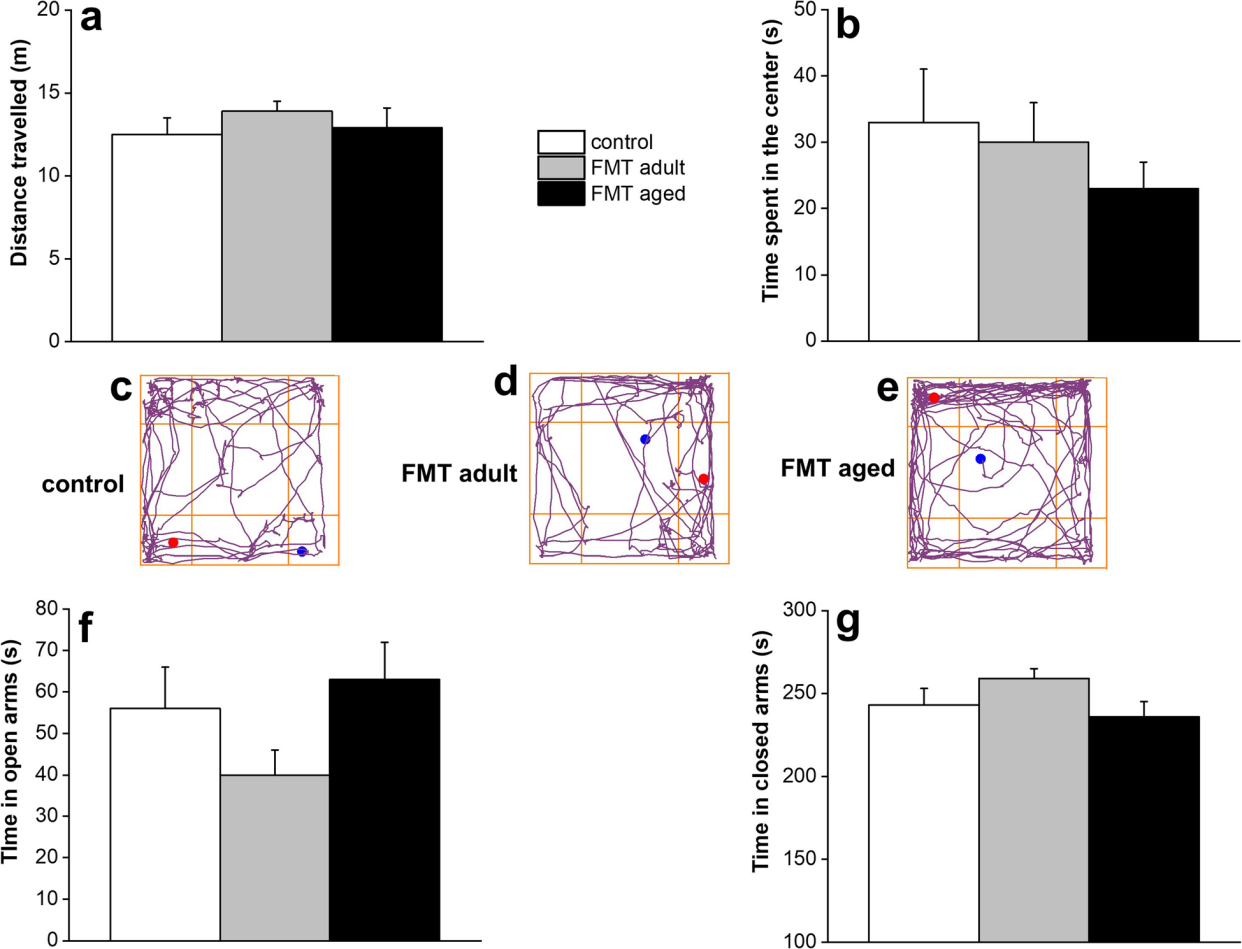
Effect of FMT from adult and old mice on locomotor and explorative activity and anxiety-related behaviour. Open field test did not show significant difference in the distance travelled by both FMT-aged and FMT-adult was observed when compared to the untreated control group indicating no significant motor impairments of the animals (Fig. 4a). However, FMT-aged mice displayed a tendency to prefer the periphery or the corners of the arena instead of the centre (Fig. 4b). The representative tracks of movement patterns are depicted in Fig. 4c–e (ANY-maze software). Furthermore, in the elevated plus maze, FMT-aged mice did not display significant differences in time spent in either arms of the maze compared to control groups (Fig. 4f, g). The values represent the mean ± SEM for each group (*n* = 10–12 mice/group)

Given that the microbiota has been reported to affect locomotor activity and anxiety-like behaviour in animal models [5, 21], we then assessed whether FMT from aged animals to adult recipients could also affect these behavioural manifestations. For this purpose, we employed two validated tests: the open field and the elevated plus maze. In the open field task, no significant difference in the distance travelled by both FMT-aged and FMT-adult was observed when compared to the untreated control group indicating no significant motor impairments of the animals (Fig. 6a). However, it is worth noting that FMT-aged mice displayed a tendency to prefer the periphery or the corners of the arena instead of the centre (Fig. 6b). The representative tracks of movement patterns are depicted in Fig. 6c–e (ANY-maze software). In the elevated plus maze, FMT-aged mice did not display significant differences in time spent in either arms of the maze compared to control groups (Fig. 6f–g) with most of the animals spending the majority of the time in the closed arms irrespective of the FMT treatment.

### FMT from aged donors alters protein expression in the hippocampus of adult recipients

The observation that FMT from aged donors to adult recipients could alter behavioural patterns of transplanted mice prompted us to investigate the molecular mechanisms underlying such changes. To this extent, a one-shot label-free quantitative proteomics approach was employed, targeting the hippocampus, a brain region known to play an important role in learning and memory and to undergo several changes with the advancing of age [22]. The analyses resulted in the quantitation of 2180 protein groups and 16,083 unique peptides (Additional file 2). The Pearson correlation coefficients between samples and technical replicates were 0.99, indicating a high reproducibility and confidence of the resulting data (Additional file 3 a). The volcano plot was obtained by two-sided *t* test of the two groups: FMT aged faeces into adult mice related to the adult FMT into adult mice (Additional file 3 b). One hundred forty proteins were differentially regulated, 52 overexpressed and 88 down-expressed, of which 47 proteins were differentially regulated with a fold change higher than 1.5 (Table 1). Furthermore, western blot analyses of two selected proteins implicated in the behavioural phenotype of transplanted mice, the microtubule-associated protein MAPT (FMT-aged versus FMT-adult ratio: 2.38) (Additional File 4) and tRNA-splicing ligase RtcB homolog Rtcb (FMT-aged versus FMT-adult of 0.46) (Additional File 5) confirmed the patterns identified by the label-free proteomics approach.

**Table 1:** Ingenuity pathway analysis (IPA)

	Functions annotation	***P*** value	Activation ***z***-score	Molecules	# molecules
Cell-to-cell signalling and Interaction, nervous system development and function	Synaptic transmission	2.83E−11	− 1.763	AMPH, ANKS1B, CAMK2A, CNP, CPNE6, DLG2, DLG4, DPYSL2, ERC2, FBXO2, GNAI2, HNRNPK, MAPT, NAPA, NPTX1, NRCAM, NSF, PAFAH1B1, PARK7, PPP3CA, PPP3R1, PRKCG, PSMC5, RAB3A, S100B, H3GL2, SLC12A5, SLC1A3, SNAP25, SNPH, SYN1, SYN2, SYNGAP1, UNC13A, VDAC1	35
Behaviour	Learning	2.72E−14	− 1.697	ACTG1, AMPH, ATP1A3, CAMK2A, CFL1, CKB, CKMT1A/CKMT1B, CPT1C, CRMP1, CTNND2, DLG3, DLG4, ELAVL4, FBXO2, GMFB, GSK3B, HAPLN1, KCNAB2, MAPT, NCAM1, NCDN, NRAS, NRCAM, NTRK2, PAFAH1B1, PARK7, PDE1B, PEX5L, PPP3CA, PPP3R1, PRKAR1A, PRKAR2B, PRKCG, RTN4, S100B, SHANK1, SLC12A5, SNAP25, SOD2, SRCIN1, SYNGAP1, SYNJ1, SYNPO, TRIM3, TSN, VDAC1	46
Cell-to-cell signalling and interaction, nervous system development and function	Neurotransmission	4.44E−12	− 1.656	AMPH, ANKS1B, CAMK2A, CNP, CPNE6, DLG2, DLG4, DNM1, DPYSL2, ERC2, FBXO2, GDAP1, GNAI2, HNRNPK, KCTD12, MAPT, NAPA, Nefm, NPTX1, NRCAM, NSF, NTRK2, PAFAH1B1, PARK7, PPP3CA, PPP3R1, PRKCG, PSMC5, RAB3A, S100B, SH3GL2, SLC12A5, SLC1A3, SNAP25, SNPH, SRCIN1, SYN1, SYN2, SYNGAP1, UNC13A, VDAC1	41
Cell morphology, cellular assembly and organization, nervous system development and function	Elongation of neurites	4.27E−08	− 0.228	ALCAM, CAMK2A, DPYSL2, GNAS, MAPT, NTRK2, OMG, PACSIN1, PAFAH1B1, PFN1, PFN2, RAB35	12
Behaviour	Locomotion	6.83E−07	− 0.087	ABAT, AGAP2, ATP1A1, ATP1A3, CAMK2A, CNP, DLG3, DLG4, DNM1, ELAVL4, GMFB, HINT1, MAPT, NCAM1, NEFL, NRCAM, NTRK2, OMG, OXR1, PAFAH1B1, PARK7, PDE1B, RTN4, SNAP25, SOD1, SOD2, SPTBN4, TSN	28

Ingenuity pathway analysis (IPA) of the significantly up- and downregulated proteins (after Bonferroni correction) in the hippocampus of young mice transplanted with faeces from aged donors (FMT-aged) versus young mice transplanted with faeces transplant from young age-matched mice (FMT-adult). The enriched categories, related to specific function annotations, the *P* value, the *z*-score of the software and the involved proteins are displayed

**Fig. 7:**
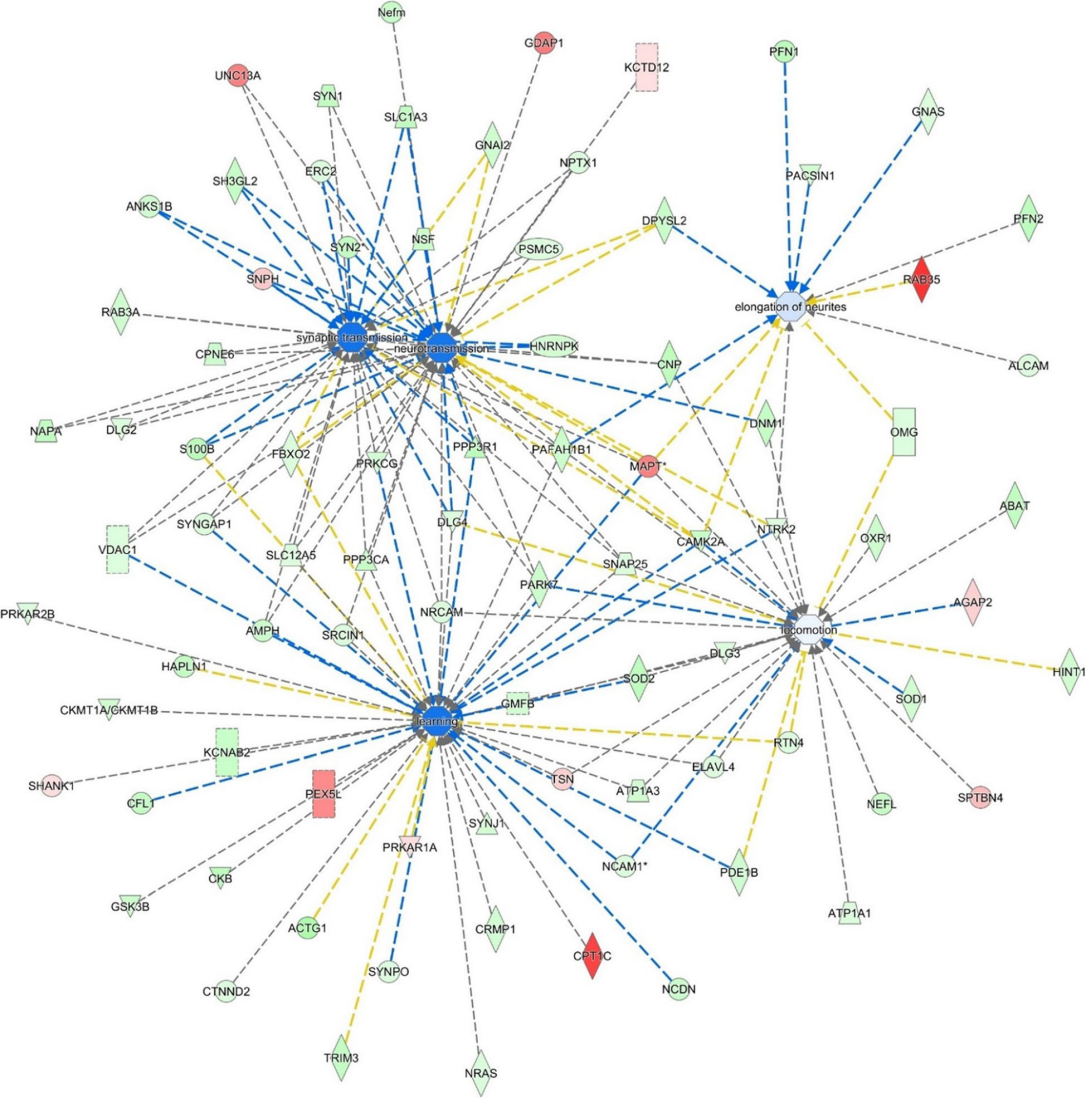
Ingenuity pathway analysis (IPA). IPA of the significantly up- and downregulated proteins (after Bonferroni correction) for faeces from aged donors transplanted in adult mice versus faeces from adult donors transplanted in adult age-matched mice, in the hippocampus tissue. The circles represent the main network node and the blue colour the significantly downregulated nodes. The upregulated proteins are marked red, while those that that were downregulated are marked in green. The intensity of the colour relates to fold-change (light to dark colour = small to large fold change). The symbols shown in the network are explained at http://www.qiagenbioinformatics.com/products/ingenuity-pathway-analysis

Subsequent protein network analyses highlighted several differentially expressed proteins in the hippocampus of adult mice that received microbiota from aged donors compared to adult mice that received microbiota from age-matched adult donors. Table 1 summarizes the main enriched categories. Cellular signalling during nervous system development and related to synaptic transmission was described by 35 molecules, differentially expressed, indicating a downregulation during the transplant (*z*-score − 1.7). Neurotransmission was also found downregulated by 41 differentially expressed molecules. Remarkably the changes in the expression of a total of 87 learning-related proteins and cognition tests in FMT-aged mice pointed to a decline of brain functions as seen during the physiological ageing process (Fig. 7). Original data for the cognition and behaviour tests can be found in Additional file 6. As a corollary, the extent of the effects of the shifts of microbiota on health and disease in ageing is further stressed by the observation that a large set of proteins involved in lipid metabolism were downregulated (Additional file 7). This latter evidence suggested that shifts of microbiota might underpin the detrimental effects of ageing on multiple health-critical functions. Thus, the single label-free run approach resulted in an in-depth hippocampus protein characterization and above all in the identification of differentially regulated proteins involved in key pathways of ageing-related processes.

**Fig. 8:**
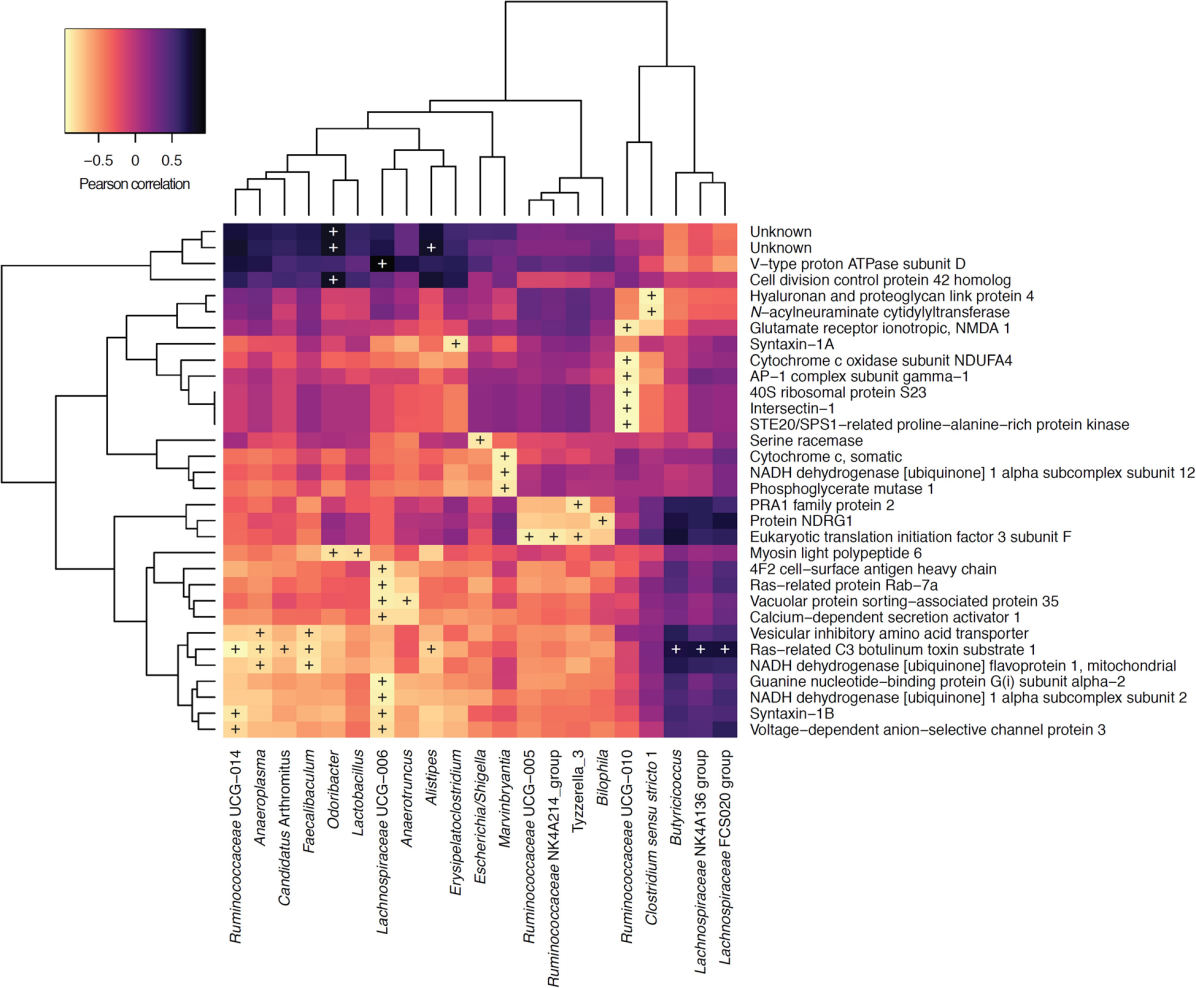
Pearson correlation of faecal microbiomic and proteomic (hippocampus) data. Only rows/columns that contained significant data (*P* < 0.1, Benjamini-Hochberg) are shown

Integration of microbiomic and proteomic data revealed few significant associations (Fig. 8). In particular, *Faecalibaculum*, *Lachnospiraceae* and *Ruminococcaceae* were found to be significantly correlated with proteins implicated in mitochondrial energy metabolism (electron transport chain, complex 1 biogenesis, oxidative phosphorylation), but also neurotransmitter transport (Fig. 8).

Furthermore, integration of metabolomic and proteomic data showed a significant association between five of the 67 metabolites and any of the 1648 hippocampal proteins (Benjamini-Hochberg (BH); *P* value < 0.05). In particular, “Guanosine derivate” was significantly correlated with 256 proteins (Additional file 8), “Arabinose” was significantly correlated with 312 proteins (Additional file 9), and “Fatty acids C6-onwards” was significantly correlated with 148 proteins (Additional file 10). “Glycolate” was only significantly anti-correlated with haemoglobin subunit beta-1 (*r* − 0.94, BH *P* value 0.031). “4-Hydroxyphenyl compound” was only significantly correlated with alpha-aminoadipic semialdehyde dehydrogenase (*r* 0.94, BH *P* value 0.025) (Additional file 11).

### FMT from aged donors into adult recipients triggers phenotypic changes in glia cells of the hippocampus fimbria but did not affect gut permeability or levels of systemic and brain cytokines

**Fig. 9:**
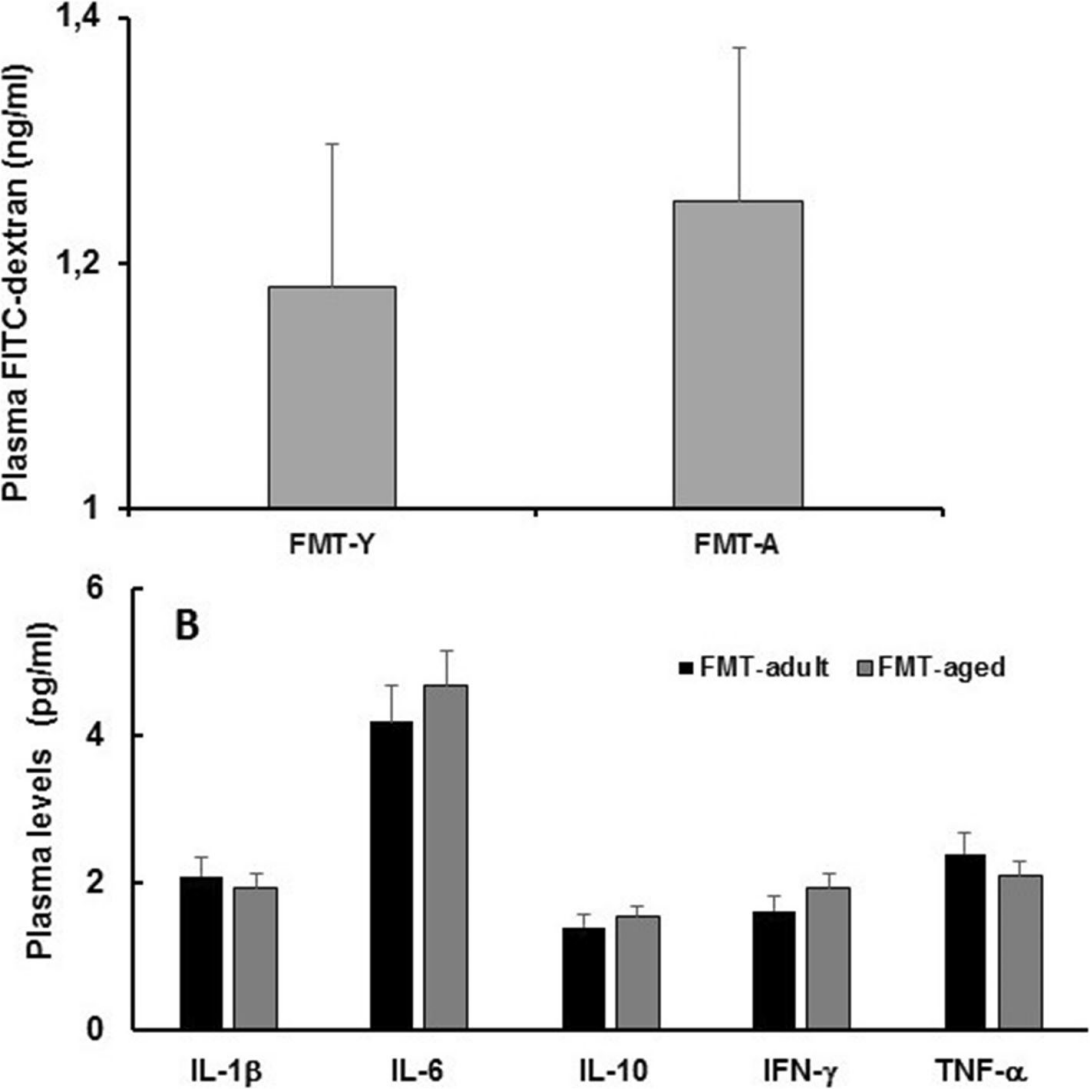
FMT from aged donors does not affect either gut permeability or circulating cytokines. Mice were orally administered with a solution of FITC-dextran and plasma levels measured after 180 min. No differences were observed between FMT-adult and FMT-aged recipients. Plasma samples were also used to evaluate levels of circulating cytokines; also in this case we failed to observe any significant change of circulating pro- and anti-inflammatory cytokines in both group of FMT-treated mice (*n* = 8 mice/group)

**Fig. 10:**
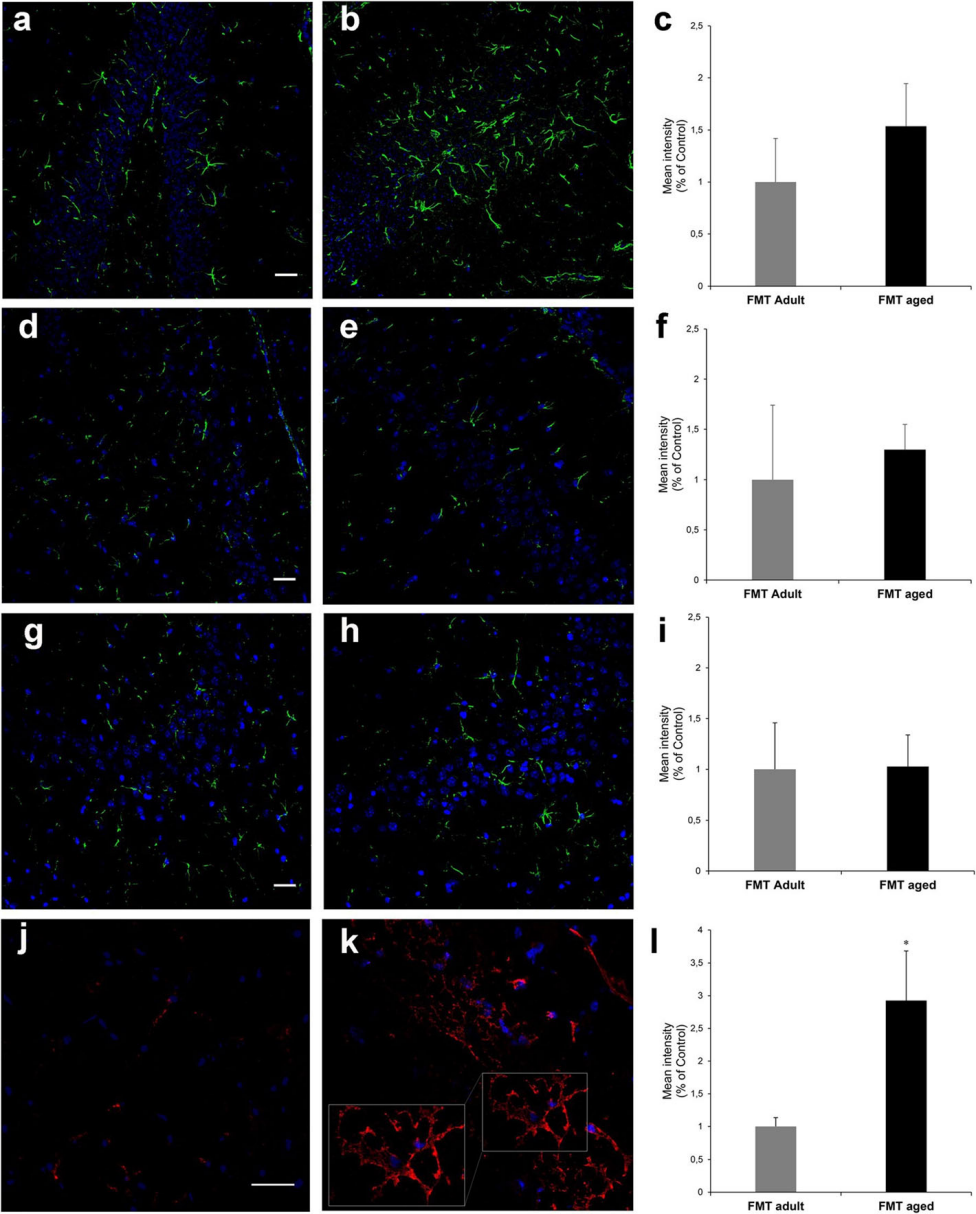
Post-FMT levels of GFAP and F4/80 in hippocampal areas. Representative images acquired at the confocal microscope with anti-GFAP antibody (green) and the relevant fluorescent intensity in different areas of the hippocampus (**a**–**h**). No difference was observed between FMT-adult (left panels **a**, **d** and **g**) and FMT-aged (middle panels **b**, **e** and **h**) in the expression of GFAP. The analysis was carried out in the dentate gyrus region (**a**, **b**, fluorescent intensity shown in **c**), CA4 region (**d**, **e**, fluorescent intensity shown in **f**) and CA3 region (**g**–**I**, fluorescent intensity shown in **i**). By contrast, a significant increase of the expression of F4/80 (red) (**j**, **k**, fluorescent intensity shown in **l**) was observed in the white matter of the hippocampus fimbria between FMT-adult (**j**) and FMT-aged mice (**k**). The detail of the hippocampus regions investigated are displayed in Additional File 13. Fluorescence intensity bars represent the mean ± SEM from 3 mice/group and asterisk indicates *P* = 0.0168. Nuclei have been counterstained with ToPro-3 (in blue). (scale bars 30 μm)

It has been suggested that FMT from aged donors triggers systemic inflammageing in adult recipients [23]. Consequently, we investigated whether FMT from aged donors triggered increased systemic levels of pro-inflammatory cytokines and intestinal permeability in adult recipients. We report that neither gut permeability (Fig. 9a) nor plasma levels of inflammatory cytokines (Fig. 9b) changed following FMT. Furthermore, levels of a large panel of cytokines were also evaluated in protein extracts from the hippocampi of FMT-treated groups (Additional file 12); similarly to what we observed at the systemic level, we did not detect a difference between any of the cytokine tested demonstrating the absence of FMT-triggered neuroinflammation, at least at this time point post-FMT. Also, we observed in specific regions of the hippocampus (Additional file 13) that FMT from aged donors did not induce increase in glial fibrillary acidic protein (GFAP) expression in astrocytes of hippocampus regions (Fig. 10a–h) further confirming the lack of an overt neuroinflammatory response. On the other hand, a significant (*P* = 0.0168) increase in the expression of F4/80, a typical trait of the ageing brain [24], was observed in glia cells in the white matter of the hippocampus fimbria (Fig. 10j–l). The expression of both GFAP and F4/80 was further confirmed by western blot (Additional file 14 and Additional file 15, respectively).

## Discussion

The decline of cognitive and behavioural functions in ageing is one of the most detrimental effects of ageing [25]. In particular, a deficit of hippocampal-dependent functions, including spatial learning and memory, is a distinctive trait of ageing [17]. Although the role of the gastrointestinal tract and gut microbiome on the development and function of the CNS has become evident in the past few years [2, 5, 3, 4, 6], the extent of the impact of the age-associated shits of the gut microbiota on specific functions of the CNS remained to be determined. Here we report that FMT from aged donors affects memory and spatial learning in transplanted adult mice, a phenomenon led through a differential expression of proteins involved in maintenance of synaptic plasticity and neurotransmission in the hippocampus area. These findings demonstrated that the transfer of an aged-like microbiota into young mice is sufficient to evoke similar memory and cognitive alterations to that found in aged mice and in so doing ultimately strengthening the role of the intestinal microbiota on key functions of the CNS. Indeed, although the short-term working memory is similar in young and aged mice, the latter exhibit significant deficits in spatial memory compared to young mice [26–31]. Similarly, the novel object recognition memory has been reported to decay with ageing [32–35]. Also, previous works showed increased anxiety-like behaviours associated to cognitive impairments in middle-aged and aged mice [36, 37], which could be prevented by prebiotic supplementation [37]. By contrast, we reported that other deficits triggered by the ageing process, such as locomotor activity and anxiety-like behaviour [38, 30], were not affected by the FMT procedure suggesting that age-associated shifts of microbiota tend to have a more selective impact on cognition and memory in mice.

Although the presence of specific microbial families and genera have been associated with cognitive decline, anxiety behaviours and affective disorders [39], to our knowledge, this is the first evidence showing that FMT significantly affects important cognitive functions of the CNS in ageing. Such observations prompted us to investigate the potential underlying mechanisms and whether the observed changes in cognition and behaviour may be triggered through the modification of proteins relevant for CNS function.

A label-free quantitative proteomics approach showed a significant modification of protein expression in the hippocampus area. In particular, the variation of the expression of proteins, such as D-dopachrome decarboxylase (D-DT), neuronal membrane glycoprotein M6-a (Gmp6A) and M6-b and Ras/Rap GTPase-activating protein SynGAP, was of significance. Indeed, alterations in the expression of these proteins in the CNS have been implicated in important CNS functions and disturbances, ranging from filopodium formation, synaptogenesis, learning disability, behavioural anomalies and neuronal plasticity to neurodegenerative disorders [40].

Although the major pathways affected by the FMT procedures pertained to synaptic transmission, learning and neurotransmission (Table 1), the label-free quantitative approach suggested that it is plausible that FMT from aged donors might mimic the effects of physiological ageing on other CNS structures and functions. For instance, although major alterations of the blood-brain barrier (BBB) components were not detected, we observed a downregulation of proteins involved in glucose transport across the BBB such as SlcA1 and A3 [41] following FMT from aged donors (Additional file 1) that might contribute to the dysfunctional bioenergetic system of the ageing brain [42]. Interestingly, integration of microbiomics and proteomics data revealed significant interactions with proteins involved in energy metabolism. Impaired brain glucose metabolism is known to compromise transmembrane ion transport, vesicle recycling and synaptic signalling [43, 44].

In addition to these observations, it has been suggested that changes in the microbiota are associated with increased gut permeability and systemic inflammation that may play an important role in age-associated alteration, of cognitive and behaviours in ageing [45]. With regard to inflammation, it has been described that FMT from aged donors to young germ-free recipients triggered systemic inflammageing including enhanced CD4^+^ T cell differentiation and distribution of several Th1 and Treg subsets, in particular in the systemic compartment [23]. However, the latter report fell short of measuring circulating pro-inflammatory cytokines and cognitive/behavioural functions in the recipients. In our experiments, a small non-significant increase was observed for IL-1β and TNF-α. More importantly, we failed to detect any increase of regulatory cytokines, either pro- or anti-inflammatory in the hippocampi of FMT-treated mice. Also, consistent with the lack of a significant inflammatory response is our observation that levels of GFAP did not increase in various areas of the hippocampus. However, currently, we cannot rule out a direct effect of pro-inflammatory cytokines on protein expression and function. Indeed, cytokine levels were assessed at the end of the intervention period and we do not know whether fluctuations of cytokine levels occurred at the early-stage post-FMT, thus rendering cytokine detection unlikely. This possibility still makes it plausible to hypothesize that the changes in the CNS observed here might be linked to a microbiota-associated increase, albeit minor inflammation, both locally and systemically.

Mice that received FMT from aged donors showed phenotypic change of the microglia, resident immunocompetent cells in the CNS [46] that monitor and regulate specific neuronal elements including synapses and neuronal cell bodies and in so doing they play an active role in neuronal surveillance in homeostatic conditions as well as in response to disease and injury. Indeed, similarly to what observed during physiological ageing [24], we observed a significant increase in the expression of the marker F4/80 in microglia cells of the hippocampus fimbria. Whether this phenotypic change of the microglia contributed to the changes observed in the hippocampus of mice following the FMT from aged donors remains to be determined. Indeed, the F4/80, an established macrophage marker, is a member of the epidermal growth factor (EGF)-transmembrane 7 (TM7) family, and although its increased expression has been linked to the induction of oral tolerance, its function remains unknown [47]

Furthermore, integration of faecal microbiomic and metabolomic data showed a clear association between lower levels of SCFAs and decreased representation of obligate anaerobes such as the *Lachnospiraceae* and *Ruminococcaceae* that have been associated with the production of SCFAs by the human faecal microbiota (Figs. 2. and 3). It should, however, be noted the biological relevance of increased levels of faecal propionate, butyrate and isobutyrate being significantly correlated with increased relative abundance of lactic acid bacteria such as *Staphylococcus*, *Jeotgalicoccus* and *Facklamia* is currently unknown (Figs. 2. and 3) as our knowledge as to the ability of these species to produce SCFAs other than acetate or lactate from fermentation of carbohydrate or protein sources is poor as is our knowledge on cross-feeding potential of the mouse gut microbiota.

In addition to these aforementioned genera, a strong decrease in *Prevotellaceae* was also observed following FMT from aged donors in adult recipients. We previously reported higher levels of *Prevotellaceae* and *Ruminococcaceae* in *APOE3/E3* and *APOE2/E3* genotype carriers, respectively, relative to *APOE4* carriers [48], one of the strongest prevalent risk factors for neuropathology and Alzheimer’s disease [49]. While the under-representation of *Prevotellaceae* has been previously reported to diminish the levels of health-promoting neuroactive SCFAs in humans [50] and the biosynthesis of thiamine and folate, two vitamins decreased in Parkinson’s disease [51, 52], depletion of *Ruminococcaceae* has been associated with Alzheimer’s disease [53]. Interestingly, a previous study showed that SCFAs may regulate host serotonin biosynthesis [54], a multifaceted neurotransmitter modulating cognition, learning and memory, along with numerous physiological processes [55]. Finally, the role of the vagus nerve on microbiota-mediated alterations of the hippocampus area cannot be ruled out. This cranial nerve exerts an important role in gut-brain communication [56], and it has been shown that it mediates the effects of orally delivered probiotic strains on aspects of behaviour [57]. Future experiments of selective vagotomy will help to elucidate the role of the vagus nerve in the microbiota-mediated decline of spatial and learning memory.

## Conclusion

Overall, these results further highlight the paramount importance of events taking place in the GI-tract in health and disease and stressed the paramount importance of the intestinal microbiota on the gut-brain axis. In particular, these demonstrated a direct impact of age-associated shifts of the intestinal microbiota composition on CNS-specific pathways that led to a significant decline of key functions, such as spatial learning and memory. This notion, along with the recently collected evidence that correcting age-associated shift of microbiota profile is beneficial for health and life expectancy [58]. Indeed, it provides a solid support to the hypothesis that microbe-based approaches that aim to restore a young-like microbiota might improve cognitive function and in so doing the quality of life of the elderly, an ever-increasing demographic segment of modern societies.

## Methods

### Animals

Adult (3 months) and aged (24 months) male C57BL/6 mice were used. Mice (Envigo, Varese, Italy or Charles River, UK) were provided with food and water ad libitum. Environmental temperature was kept at 23 ± 1 °C with a 12-h light/dark cycle. All efforts were made to minimize animal suffering and to reduce the number of mice used.

### Faecal material preparation and FMT regime

Faecal material was collected from adult and aged mice and placed into Eppendorf tubes containing 500 mL of freezing solution (sterile saline solution with 12.5% glycerol) and homogenized. The suspended pellets were then stored at − 80 °C until utilized. For FMT, mice were randomized into the following groups (*n* = 12 per group): control (no antibiotic treatment, no FMT), FMT-adult (antibiotic treatment followed by FMT from adult age-matched donors) and FMT-aged (antibiotic treatments followed by FMT from aged donors). Antibiotic mix was administered by oral gavage. The antibiotic/anti-fungal regime (Additional file 16) was as follows: day 1–3 mice were gavaged daily with anti-fungal treatment with amphotericin B 1 mg kg-1, day 4–17 mice received a daily gavage of metronidazole 100 mg kg-1 while the antibiotic mix (ampicillin 1 g L-1, vancomycin 0.5 g L-1 and neomicin 1 g L-1 was added to drinking water), day 18-24 daily oral gavage with ampicillin 1 g L-1, vancomycin 0.5 g L-1, neomicin 1 g L-1, metronidazole 100 mg kg-1, amphotericin B mg kg-1. FMT was carried out via oral gavage with a faecal suspension (100 mg mL-1) in a final volume of 150 mL. FMT was performed six times on days 24–28 and 35 from the beginning of antibiotic regime.

### DNA extraction, amplicon sequencing and analyses of 16S rRNA gene sequence data and metabolomics analysis

Pre- and post-FMT faecal material was used for microbiota profiling. DNA was extracted from faecal samples using the FastDNA SPIN Kit for Soil (MP Biomedicals) with three bead-beating periods of 1 min as previously described [59]. DNA concentration was normalized to 1 ng/μL by dilution with DNA elution solution (MP Biomedicals, UK) to produce a final volume of 20 μL. Normalized DNA samples were sent to the Earlham Institute (Norwich, UK) for PCR amplification of 16S rRNA genes and paired-end Illumina sequencing (2 × 250 bp) on the MiSeq platform. The V4 hypervariable region of the 16S rRNA genes was amplified using the 515F and 806R primers with built-in degeneracy as previously reported [60, 61]. Sequence data were provided in fastq format. All processing and analyses were done in R/Bioconductor making use of the following packages: GEOquery 2.50.0 [62], dada2 1.10.0 [63], phyloseq 1.26.0 [64], tidyverse 1.2.1 (https://www.tidyverse.org), vegan 2.5.3, viridis 0.5.1, msa 1.14.0 [65], phangorn 2.4.0, ALDEx2 1.14.0 [59] and gplots 3.0.1. Taxonomy was assigned to chimera-free Exact Sequence Variants [66] using Silva 132 (downloaded from https://zenodo.org/record/1172783#.W-B0iS2cZBw on 5 November 2018). Data were filtered to remove undefined phyla and taxa present in fewer than two animals. Significance of differences between different diversity measures was determined using Wilcoxon rank sum test. ALDEx2 was used to determine statistically significant differences (Welch’s *t* test, Wilcoxon) between mouse groups. The 16S rRNA gene sequence data have been deposited in the NCBI BioProject database (https://www.ncbi.nlm.nih.gov/bioproject/) under accession number PRJNA524024.

Metabolites were analysed and quantified by 1H-NMR analysis as previously described [67]. Briefly, 20 mg of frozen faeces was thoroughly mixed on a vortex with 1 mL of saline phosphate buffer followed by centrifugation (18,000 g, 1 min). High-resolution 1H-NMR spectra were recorded on a 600-MHz Bruker Avance spectrometer fitted with a 5-mm TCI proton-optimized triple resonance NMR “inverse” cryoprobe and a 60-slot autosampler (Bruker, Rheinstetten, Germany). Metabolites were identified using information found in the literature or on the Human Metabolome Database, http://www.hmdb.ca/ and by use of the 2D-NMR methods, (e.g. COSY, HSQC and HMBC [68]) and quantified using the software Chenomx® NMR Suite 7.0TM.

### Behavioural tests

The tests were performed following the order of description and between 08:00 am and 03:00 pm. Barnes Maze test: this test was conducted according to previously described method [69]. All sessions were performed on a wet platform under a room lightning of 400 lux to increase the mouse aversion for the platform. Sessions were recorded using a video-tracking system (ANY-maze, Ugo Basile, Varese, Italy). The platform and the escape box were cleaned thoroughly between each mouse session and the surface of the platform was moistened again to avoid mice using olfactory cues to solve the task. Before performing the test, mice were trained to find the cage for 4 days with 2 sessions per day. Open field test (OFT) was conducted as previously described [70]. Briefly, mice were placed in the centre of the OFT and the total distance the mice travelled along with the time they spent in the centre of the field within 5 min was recorded with a video tracking system. The open field maze was cleaned between each mouse with 20% ethanol to eliminate odour. Novel object recognition test was carried out as previously described [71]. For each mouse, the time spent interacting with each object during the acquisition and recognition phases was video recorded and analysed blind. The corrected mnesic index (or discrimination index) was calculated as follows: (time spent exploring the novel object − time spent exploring the well-known object)/total time spent exploring both the objects. To evaluate anxiety-related behaviour, the elevated plus-maze test was conducted as previously described [38]. Time spent in each kind of arms (s) was recorded for 5 min. Percentage of entries into the open arms and that of time spent in the open arms were determined. Data acquisition and analysis were performed automatically using ANY-maze software.

### Liquid chromatography-MS analysis

Each sample was analysed by nLC MS/MS Orbitrap Fusion trihybrid mass spectrometer coupled with a nano flow UHPLC system (Thermo Fischer Scientific, USA), via a nano electrospray source with an ID 0.01 mm fused silica PicoTip emitter (New Objective). The peptides were separated, after trapped on a C18 pre-column, using a gradient of 3–40% acetonitrile in 0.1% formic acid, over 50 min at flow rate of 300 nL/min, at 40 °C. The MS method consisted of a full scan in Orbitrap analyser (120,000 resolution), followed by the combination of CID and HCD collisions. The peptides were fragmented in the linear ion trap by a data-dependent acquisition method, selecting the 40 most intense ions. Dynamic exclusion of sequenced peptides was set to 30 s. Data were acquired using Xcalibur software (Thermo Scientific, USA). All analyses were performed in triplicate. MS raw data were analysed by MaxQuant (version 1.6.2.3), using Andromeda search engine in MaxQuant and consulting the Homo Sapiens UniProtKB database; the tolerance on parents was 10 ppm and on fragments was 0.02 ppm. The variable modifications allowed were oxidation on methionine and acetylation on N terminus and carbamidomethylation on cysteine as fixed modification. The false discovery rate was below 1%, using a decoy and reverse database, and a minimum number of seven amino acids were required for peptide identification. Proteins and protein isoforms were grouped into protein groups. Label-free quantitative analyses were also performed by MaxQuant software, using the MaxLFQ algorithm. The quantitation values were obtained on high-resolution three-dimensional peptide profiles in mass-to-charge, retention time and intensity space. The mass spectrometry proteomics data have been deposited to the ProteomeXchange Consortium via the PRIDE [72] partner repository with the dataset identifier PXD016432.

### Intestinal permeability and plasma cytokines

Plasma levels of cytokines were assessed by enzyme-linked immunosorbent assays (ELISA). Assays were carried out according to the manufacturer’s instructions (all kits from eBioscience). Also, the level of cytokines was determined in protein extracts from hippocampi of FMT-treated mice using Procarta Plex Analyst 1.0 (eBioscience) following instructions provided by the manufacturer. Intestinal permeability was assessed using a method described previously [73]. Animals were orally delivered with 0.5 mL of PBS containing 25 mg of FITC-labelled dextran (FD4; Sigma-Aldrich), and blood samples were collected after 45 min. Plasma concentration of FD4 was assessed using fluorescence spectrometer (LS 55 conducted with FL WinLab software, PerkinElmer, USA) at an excitation wavelength of 490 nm and emission wavelength of 520 nm.

### Statistical analyses

Microbiome data (genus level) were correlated (Pearson) with metabolomic and proteomic data using aldex.corr() within the Bioconductor package ALDEx2 [59]. For the MS study, all data were evaluated by Perseus statistical software. The protein expression fold change variation between two groups was analysed by two sides *t* test, setting FDR less than 0.015 and s0 of 0.1. The differentially expressed proteins, with a significant ratio FMT-aged/FMT-adult (*P* value < 0.05), were analysed by Ingenuity Pathway Analyses (Qiagen), and only the differentially regulated pathway with a significant *z*-score, after Bonferroni test, was considered. The symbols shown in the network are explained at http://www.qiagenbioinformatics.com/products/ingenuity-pathway-analysis. Behavioural measurements were performed for each treatment in two different experiments (*n* = 10–12 mice/group). All assessments were performed in blind of the treatment received by the mouse groups. Results were expressed as means ± S.E.M. and the analysis of variance was performed by two-way ANOVA. A Bonferroni’s significant difference procedure was used as post hoc comparison. *P* values of less than 0.05 or 0.01 were considered significant. Data were analysed using the “Origin 9” software (OriginLab, Northampton, USA).

## Supplementary information

**Additional file 1.** Selection of donors. We carried out metabolomics analysis of the intestinal luminal contents aged (red) and young (green) mice; germ-free mice were used here as control for the metabolomic profile (blue). Using 1H-NMR to profile luminal metabolites we were able to obtain insights into microbiota metabolism and function that can only be inferred from metagenomics or transcriptomics approaches. Using Principal Component Analysis to compare and contrast 89 metabolites (including amino acids, organic acids, sugars, nucleosides, short chain fatty acids, etc.) clear separations of the metabolic profile detected in germ free (GF) and conventionalised adult and aged mice were apparent (PC1). Of particular relevance was the finding that young and aged mice housed under identical environmental conditions, were discriminated (PC2), confirming age-related changes in the profile of intestinal microbiota in mice. The aged donors displayed a higher degree of heterogeneity compared to young mice. Two donors from the aged population and three from the young cohort that were considered representative of the two populations were selected (circles) and used as donors for FMT.

**Additional file 2.** List of protein evaluated by free-label proteomics.

**Additional file 3.** Post-FMT quantitative analysis of proteins in the hippocampus. Volcano plot of quantified proteins in hippocampus tissue (A). Differentially regulated proteins due to faeces from aged mice transplanted into adult mice (T) versus faeces from adult mice transplanted into adult age-matched mice (C) are showed (T/C). The proteins in red are up regulated and in green down regulated. Scatter Plot of protein intensities (B) obtained by label free quantitation by MaxLFQ in MAxQuant, showing the Person correlation coefficients between biological and technical replicates of analysed samples.

**Additional file 4.** Western Blot analysis for MAPT in the hippocampus of FMT-treated mice. Polyacrylamide (12%) gel stained with blue Coomassie with a representative image of molecular weight marker with relevant kDa (a). GAPDH visualized bands and merged with nitrocellulose membrane (b). Mapt visualized bands and merged with nitrocellulose membrane (c). In (d) a representative histogram shows levels of analysed protein both in FMT-Y and FMT-A treated and control animals. Lane 1 (positive control, SH-SY5Y cell line); lane 2 (negative control, H292 cell line); lane 3 (aged mouse hippocampal proteins); lane 4 (adult mouse hippocampal protein); lane 5 (FMT-aged hippocampal proteins); lane 6 (FMT-adult hippocampal proteins). Mapt protein was detected approximately at 50 kDa (right blots). GAPDH (37 kDa) was used as housekeeping.

**Additional file 5.** Western Blot analysis for RTCB in the hippocampus of FMT-treated mice. Polyacrylamide (12%) gel stained with blue Coomassie with a representative image of molecular weight marker with relevant kDa (a). GAPDH visualized bands (panel b, left) and merged with nitrocellulose membrane (panel b, right). Rtcb visualized bands (panel c, left) and merged with nitrocellulose membrane (panel c, right). In (d) representative histogram shows levels of analysed protein both in FMT-Y and FMT-A treated and control animals. Lane 1 (positive control, SH-SY5Y cell line); lane 2 (negative control, mouse adipose tissue); lane 3 (aged mouse hippocampal proteins); lane 4 (adult mouse hippocampal protein); lane 5 (MT-aged hippocampal proteins); lane 6 (MT-adult hippocampal proteins). Rtcb protein was detected approximately at 56 kDa (right blots). GAPDH (37 kDa) was used as housekeeping (left panel). Molecular weight (mw) used was SHARPMASS VII.

**Additional file 6.** Raw data for the cognitive and behavioural tests (XLS file).

**Additional file 7.** Ingenuity pathway analysis (IPA). IPA analysis of the significantly up- and down-regulated proteins (after Bonferroni analysis) for faeces from aged donors transplanted in adult mice versus faeces from adult donors transplanted into adult age-matched mice, in hippocampus tissue. The circles represent the main network node and the blue colour the significantly down regulated. The up-regulated proteins are marked in red, while those that that were down-regulated are marked in green.

**Additional file 8.** Metabolome-proteome correlation (Pearson). Results for Guanosine derivate.

**Additional file 9.** Metabolome-proteome correlation (Pearson). Results for Arabinose .

**Additional file 10.** Metabolome-proteome correlation (Pearson). Results for Fatty acids C6 onwards .

**Additional file 11.** Metabolome-proteome correlation (Pearson) .

**Additional file 12.** Cytokine levels in the hippocampi.

**Additional file 13.** (Schematic of the regions of the hippocampus). Schematic frontal section of a mouse hemibrain to show the regions were confocal images were acquired to measure the GFAP and F4/80 fluorescence intensities. Images were taken from the dentate gyrus (DG), and from the CA4 and CA3 regions of the hippocampal gyrus (GFAP immunofluorescence) at -1.9 mm from the bregma. Images of the fimbria (F4/80 immunofluorescence) were acquired at -1.4 mm from the bregma. Lat ventr = lateral ventricule; cc = corpus callosum.

**Additional file 14.** GFAP western blotting. Polyacrylamide (10%) gel stained with blue Coomassie with a representative image of molecular weight marker with relevant kDa (a). GAPDH visualized bands and merged with nitrocellulose membrane (b). GFAP visualized bands and merged with nitrocellulose membrane (c). In (d) a representative histogram shows levels of analysed protein both in FMT-Y and FMT-A treated and control animals. Lane 1 (positive control, DITNC1 astrocyte-derived cell line); lane 2 (negative control, BV-2 microglial cell line); lane 3 (aged mouse hippocampal proteins); lane 4 (adult mouse hippocampal protein); lane 5 (MT-aged hippocampal proteins); lane 6 (MT-adult hippocampal proteins). GFAP protein was detected approximately at 55 kDa (right blots). GAPDH (37 kDa) was used as housekeeping (left panel). Molecular weight (mw) used was SHARPMASS VII.

**Additional file 15.** F4/80 western blotting. Polyacrylamide (8%) gel stained with blue Coomassie with a representative image of molecular weight marker with relevant kDa (a). GAPDH visualized bands and merged with nitrocellulose membrane (b). F4/80 visualized bands and merged with nitrocellulose membrane (c). In (d) a representative histogram shows levels of analysed protein both in FMT-Y and FMT-A treated and control animals. Lane 1 (Negative control, RBE4 brain endothelial cell line); lane 2 (positive control, BV-2 microglial cell line); lane 3 (aged mouse hippocampal proteins); lane 4 (adult mouse hippocampal protein); lane 5 (MT-aged hippocampal proteins); lane 6 (MT-adult hippocampal proteins). F4/80 protein was detected approximately at 160 kDa (right blots). αTubulin (52 kDa) was used as housekeeping (left panel). Molecular weight (mw) used was SHARPMASS VII.

**Additional file 16.** Summary of FMT procedure.

## Data Availability

The proteomics dataset has been submitted to ProteomeXchange via the PRIDE database (Project Name: Hippocampus proteome Mus musculus; Project accession: PXD016432). The 16S rRNA gene sequence data have been deposited in the NCBI BioProject database (https://www.ncbi.nlm.nih.gov/bioproject/) under accession number PRJNA524024. Other original data (proteins quantified and identified in the hippocampus tissue and values pertaining to cognition and behavioural tests) are included as additional files while others (metabolomics, immunohistochemistry, cytokine expression, video recording of cognitive and behavioural tests) will be made available upon request.
