# Supplementary figures and images for: Faecal microbiota transplant from aged donor mice affects spatial learning and memory via modulating hippocampal synaptic plasticity- and neurotransmission-related proteins in young recipients
Source: Microbiome. 2020 Oct 1;8:140. doi: 10.1186/s40168-020-00914-w (PMC7532115; doi:10.1186/s40168-020-00914-w)

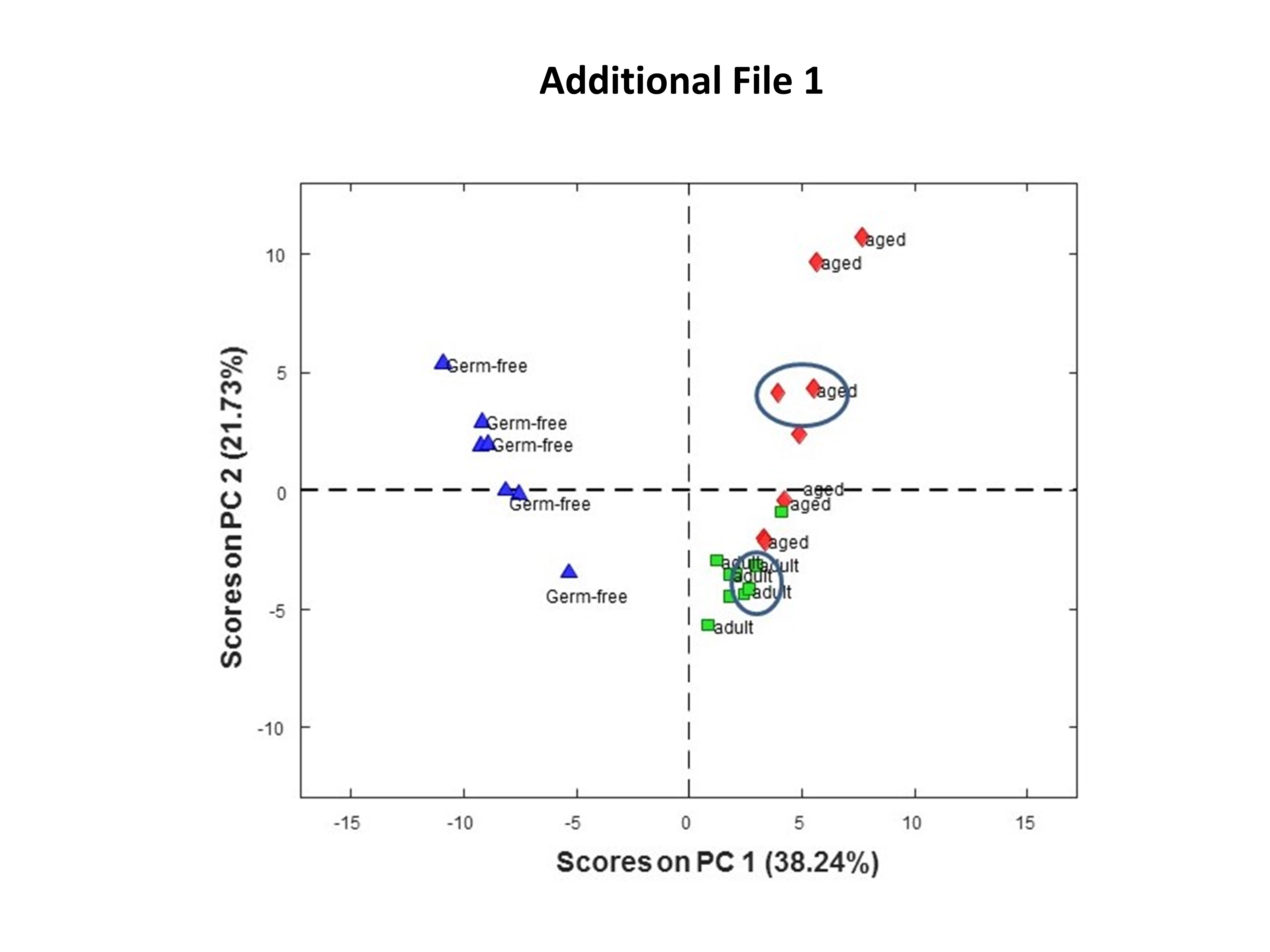

Supplement: Supplementary file 2 — Additional file 1. Selection of donors. We carried out metabolomics analysis of the intestinal luminal contents aged (red) and young (green) mice; germ-free mice were used here as control for the metabolomic profile (blue). Using 1H-NMR to profile luminal metabolites we were able to obtain insights into microbiota metabolism and function that can only be inferred from metagenomics or transcriptomics approaches. Using Principal Component Analysis to compare and contrast 89 metabolites (including amino acids, organic acids, sugars, nucleosides, short chain fatty acids, etc.) clear separations of the metabolic profile detected in germ free (GF) and conventionalised adult and aged mice were apparent (PC1). Of particular relevance was the finding that young and aged mice housed under identical environmental conditions, were discriminated (PC2), confirming age-related changes in the profile of intestinal microbiota in mice. The aged donors displayed a higher degree of heterogeneity compared to young mice. Two donors from the aged population and three from the young cohort that were considered representative of the two populations were selected (circles) and used as donors for FMT. [file 40168_2020_914_MOESM1_ESM.jpg]

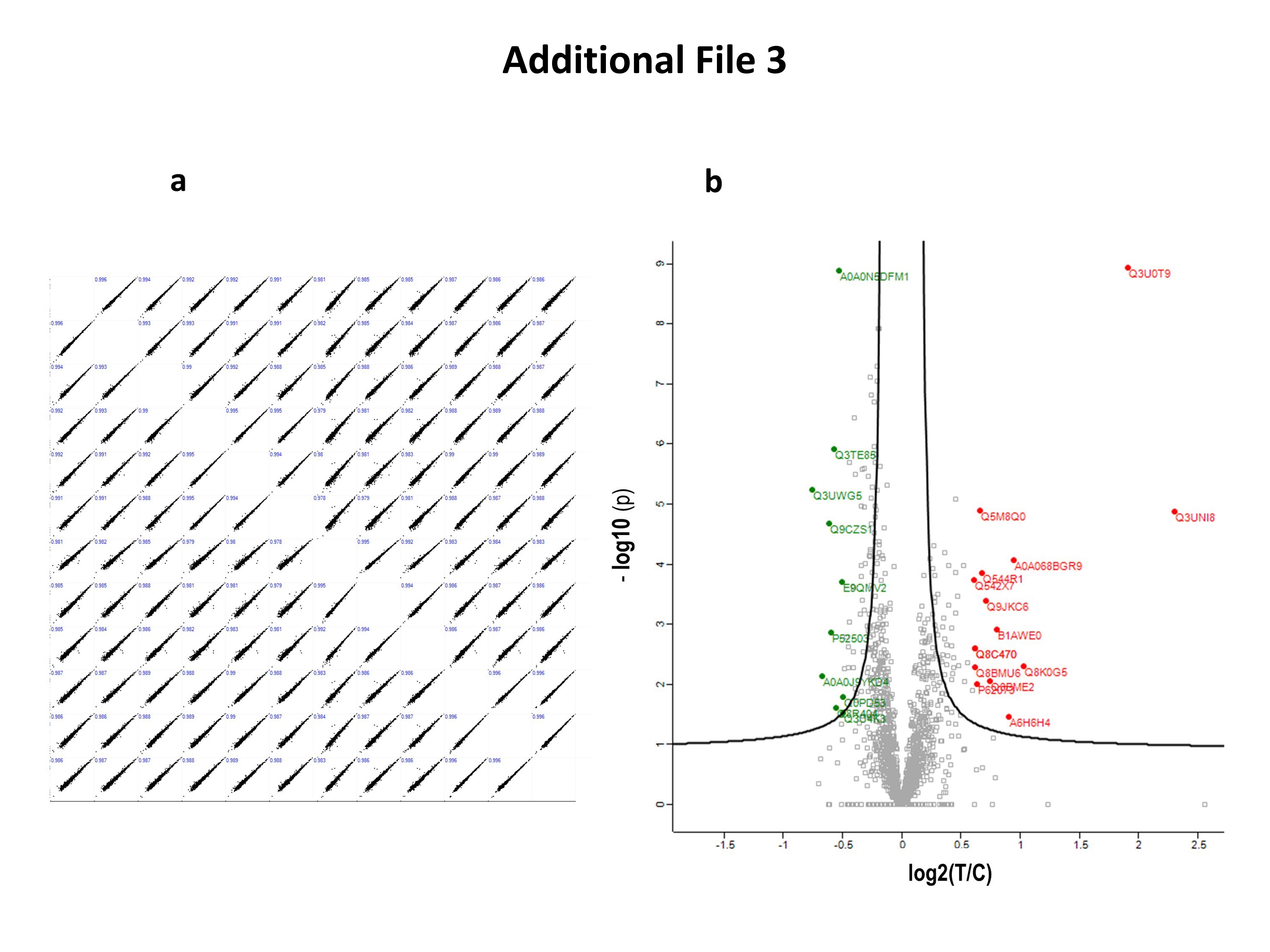

Supplement: Supplementary file 4 — Additional file 3. Post-FMT quantitative analysis of proteins in the hippocampus. Volcano plot of quantified proteins in hippocampus tissue (A). Differentially regulated proteins due to faeces from aged mice transplanted into adult mice (T) versus faeces from adult mice transplanted into adult age-matched mice (C) are showed (T/C). The proteins in red are up regulated and in green down regulated. Scatter Plot of protein intensities (B) obtained by label free quantitation by MaxLFQ in MAxQuant, showing the Person correlation coefficients between biological and technical replicates of analysed samples. [file 40168_2020_914_MOESM3_ESM.jpg]

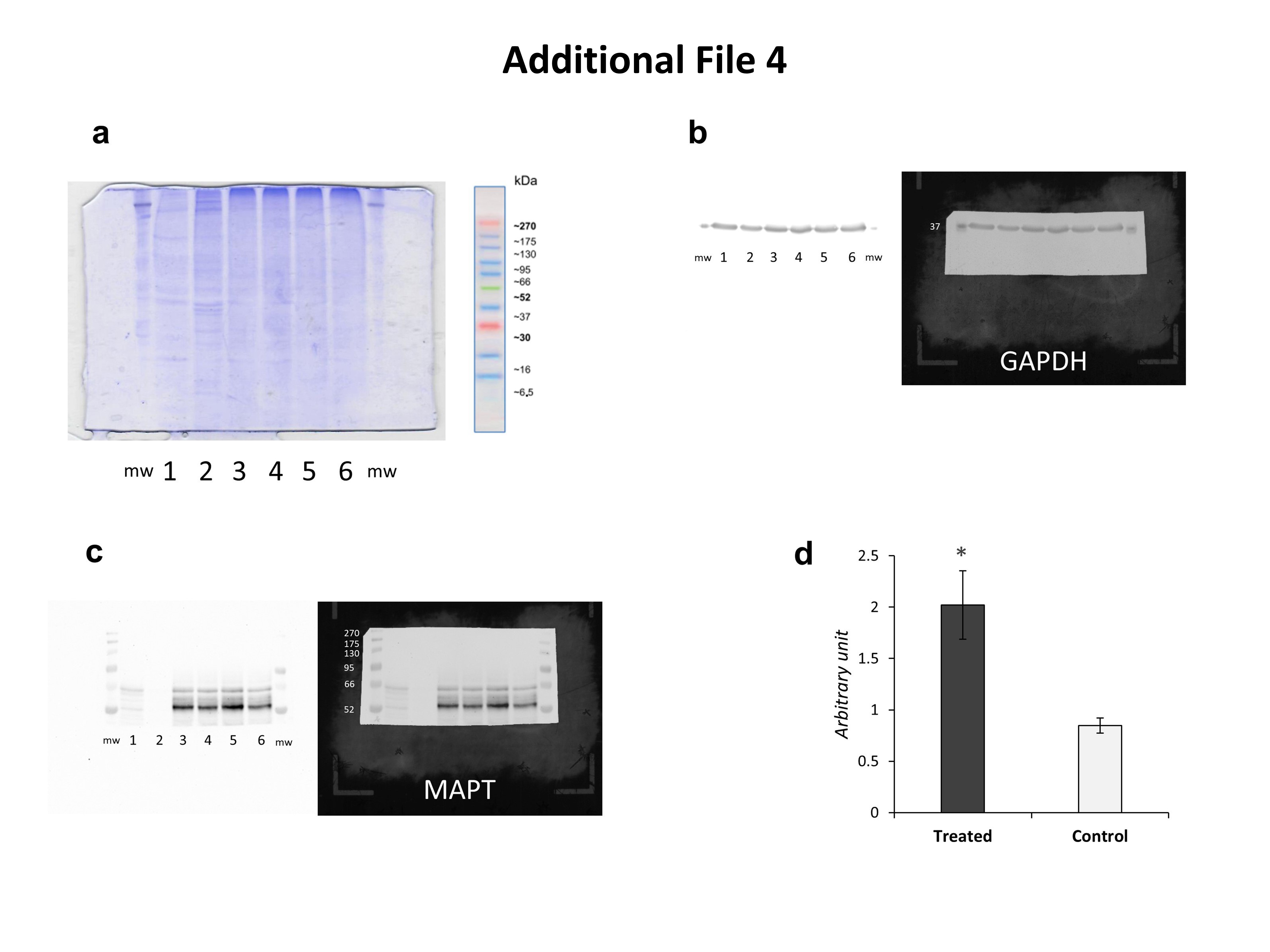

Supplement: Supplementary file 5 — Additional file 4. Western Blot analysis for MAPT in the hippocampus of FMT-treated mice. Polyacrylamide (12%) gel stained with blue Coomassie with a representative image of molecular weight marker with relevant kDa (a). GAPDH visualized bands and merged with nitrocellulose membrane (b). Mapt visualized bands and merged with nitrocellulose membrane (c). In (d) a representative histogram shows levels of analysed protein both in FMT-Y and FMT-A treated and control animals. Lane 1 (positive control, SH-SY5Y cell line); lane 2 (negative control, H292 cell line); lane 3 (aged mouse hippocampal proteins); lane 4 (adult mouse hippocampal protein); lane 5 (FMT-aged hippocampal proteins); lane 6 (FMT-adult hippocampal proteins). Mapt protein was detected approximately at 50 kDa (right blots). GAPDH (37 kDa) was used as housekeeping. [file 40168_2020_914_MOESM4_ESM.jpg]

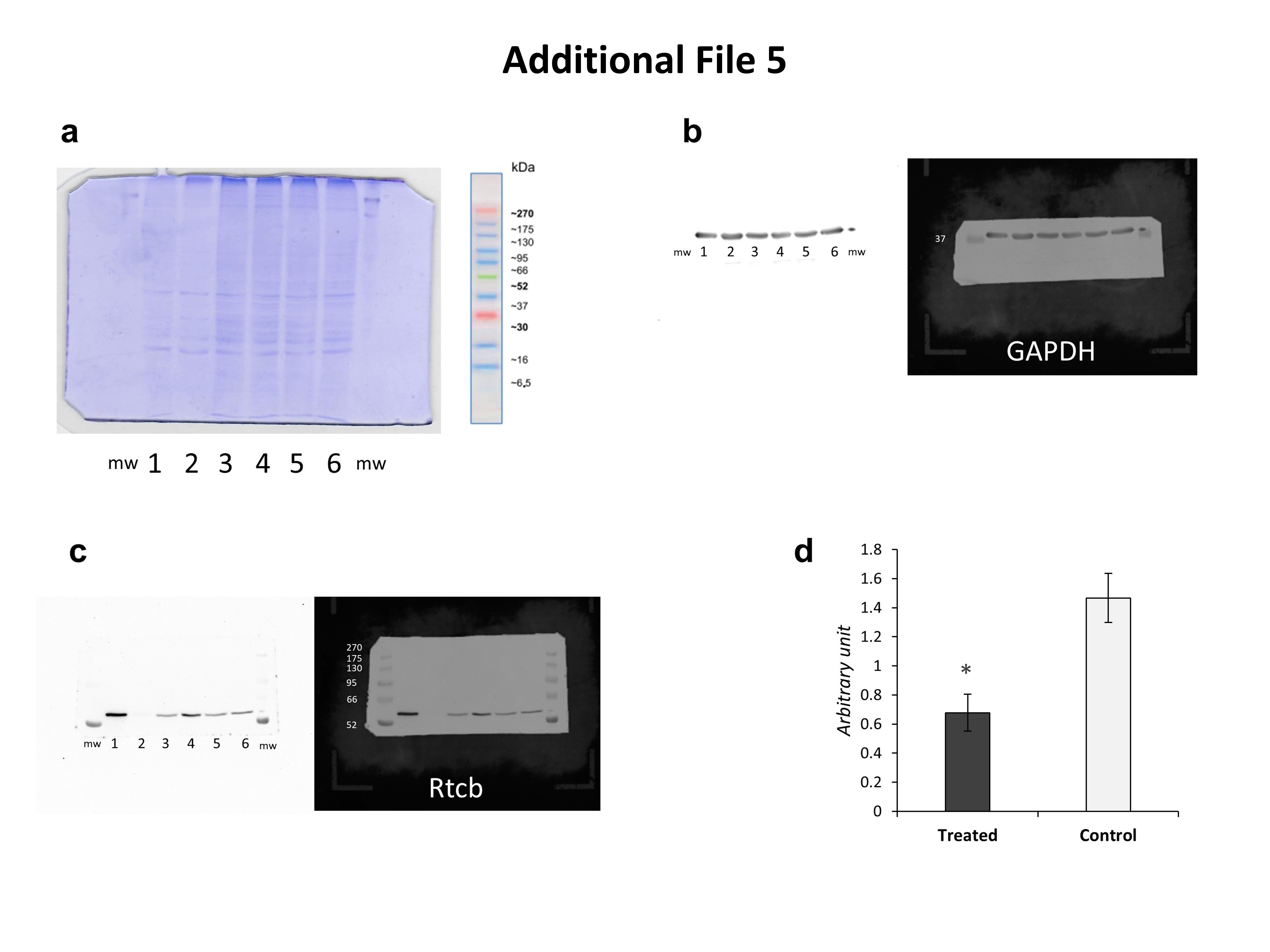

Supplement: Supplementary file 6 — Additional file 5. Western Blot analysis for RTCB in the hippocampus of FMT-treated mice. Polyacrylamide (12%) gel stained with blue Coomassie with a representative image of molecular weight marker with relevant kDa (a). GAPDH visualized bands (panel b, left) and merged with nitrocellulose membrane (panel b, right). Rtcb visualized bands (panel c, left) and merged with nitrocellulose membrane (panel c, right). In (d) representative histogram shows levels of analysed protein both in FMT-Y and FMT-A treated and control animals. Lane 1 (positive control, SH-SY5Y cell line); lane 2 (negative control, mouse adipose tissue); lane 3 (aged mouse hippocampal proteins); lane 4 (adult mouse hippocampal protein); lane 5 (MT-aged hippocampal proteins); lane 6 (MT-adult hippocampal proteins). Rtcb protein was detected approximately at 56 kDa (right blots). GAPDH (37 kDa) was used as housekeeping (left panel). Molecular weight (mw) used was SHARPMASS VII. [file 40168_2020_914_MOESM5_ESM.jpg]

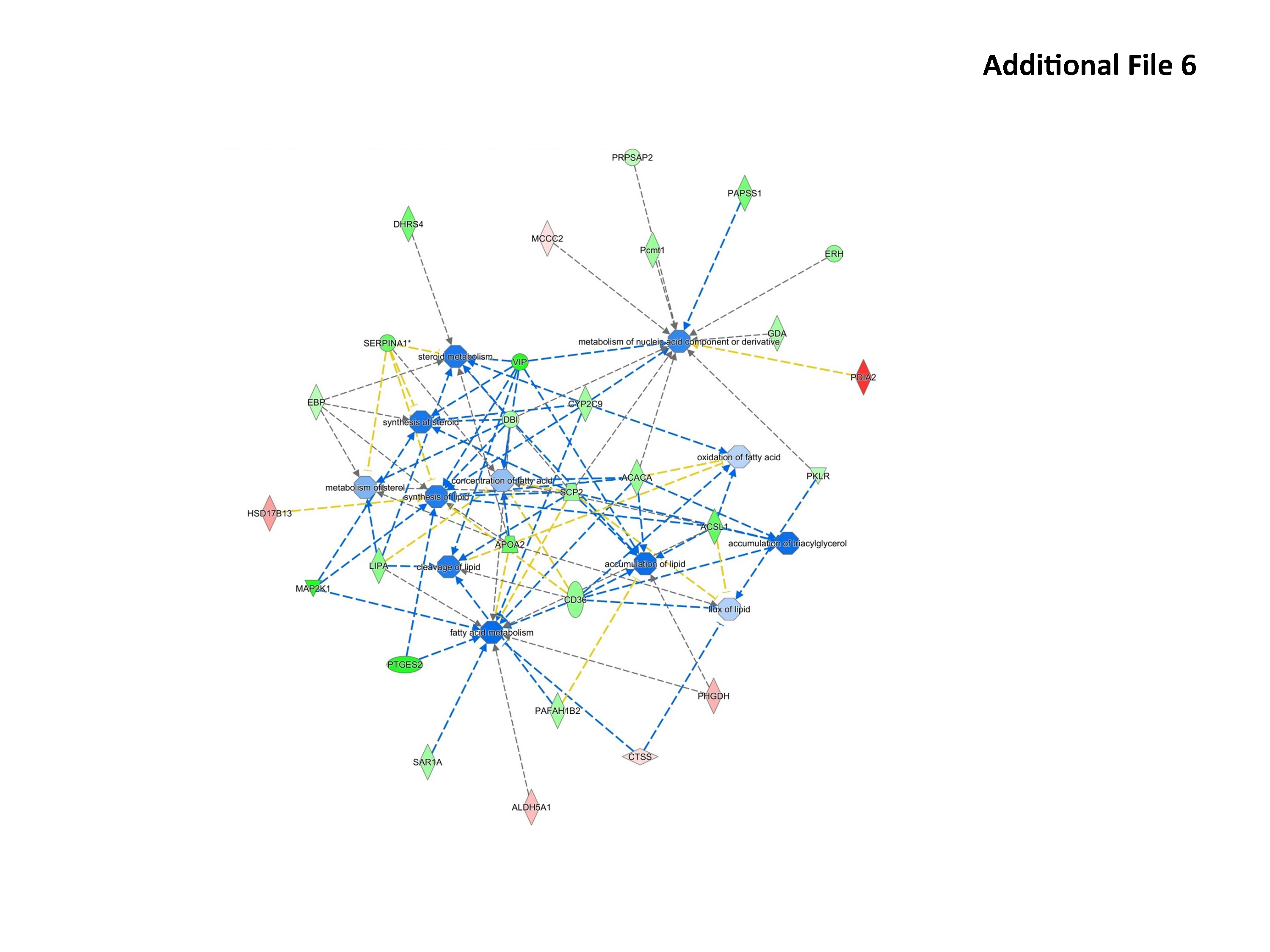

Supplement: Supplementary file 8 — Additional file 7. Ingenuity pathway analysis (IPA). IPA analysis of the significantly up- and down-regulated proteins (after Bonferroni analysis) for faeces from aged donors transplanted in adult mice versus faeces from adult donors transplanted into adult age-matched mice, in hippocampus tissue. The circles represent the main network node and the blue colour the significantly down regulated. The up-regulated proteins are marked in red, while those that that were down-regulated are marked in green. [file 40168_2020_914_MOESM7_ESM.jpg]

Guanosine derivative

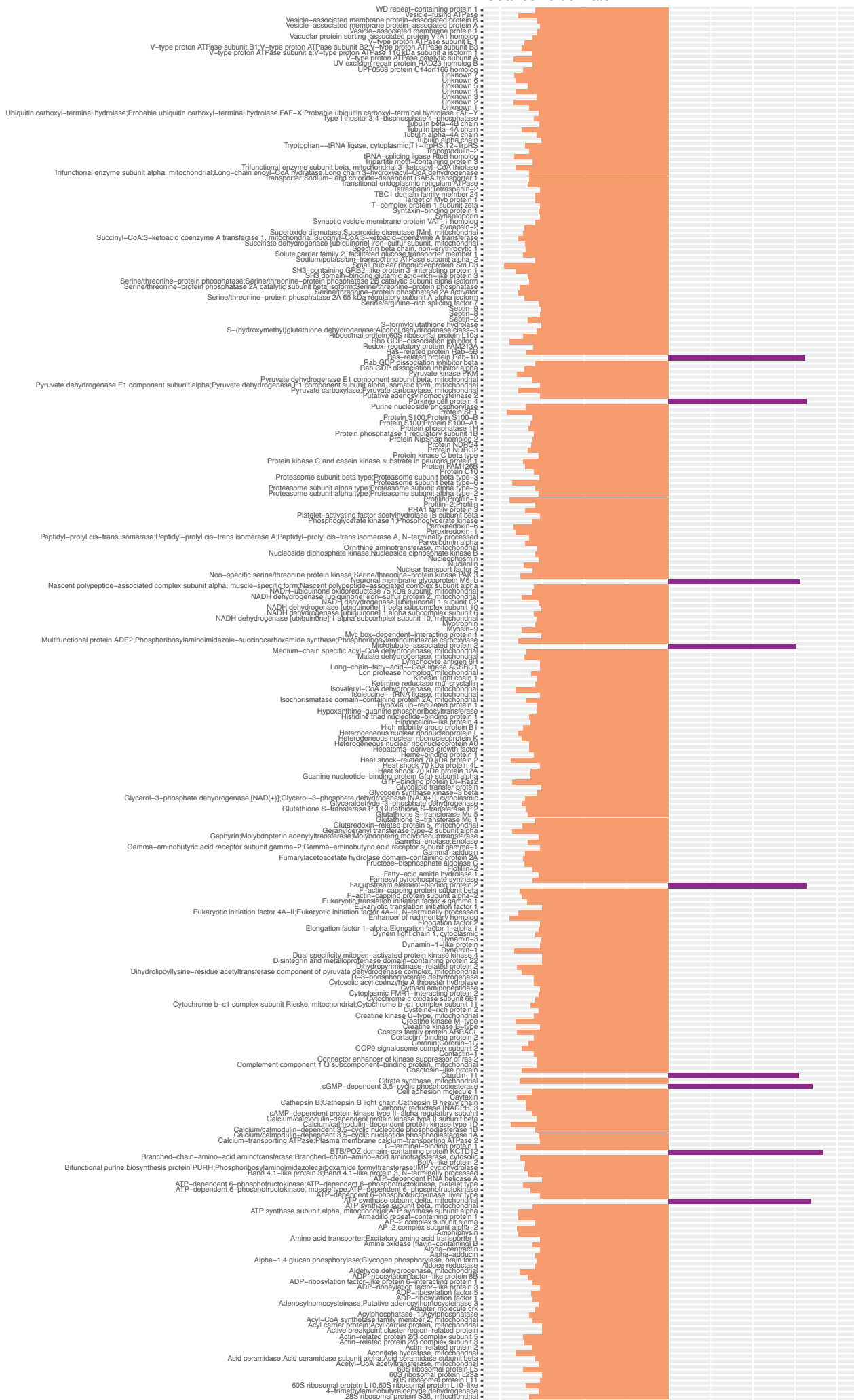

Pearson correlation (sig.  $P < 0.05$ , BH)

Supplement: Supplementary file 9 — Additional file 8. Metabolome-proteome correlation (Pearson). Results for Guanosine derivate. [file 40168_2020_914_MOESM8_ESM.pdf]

Arabinose

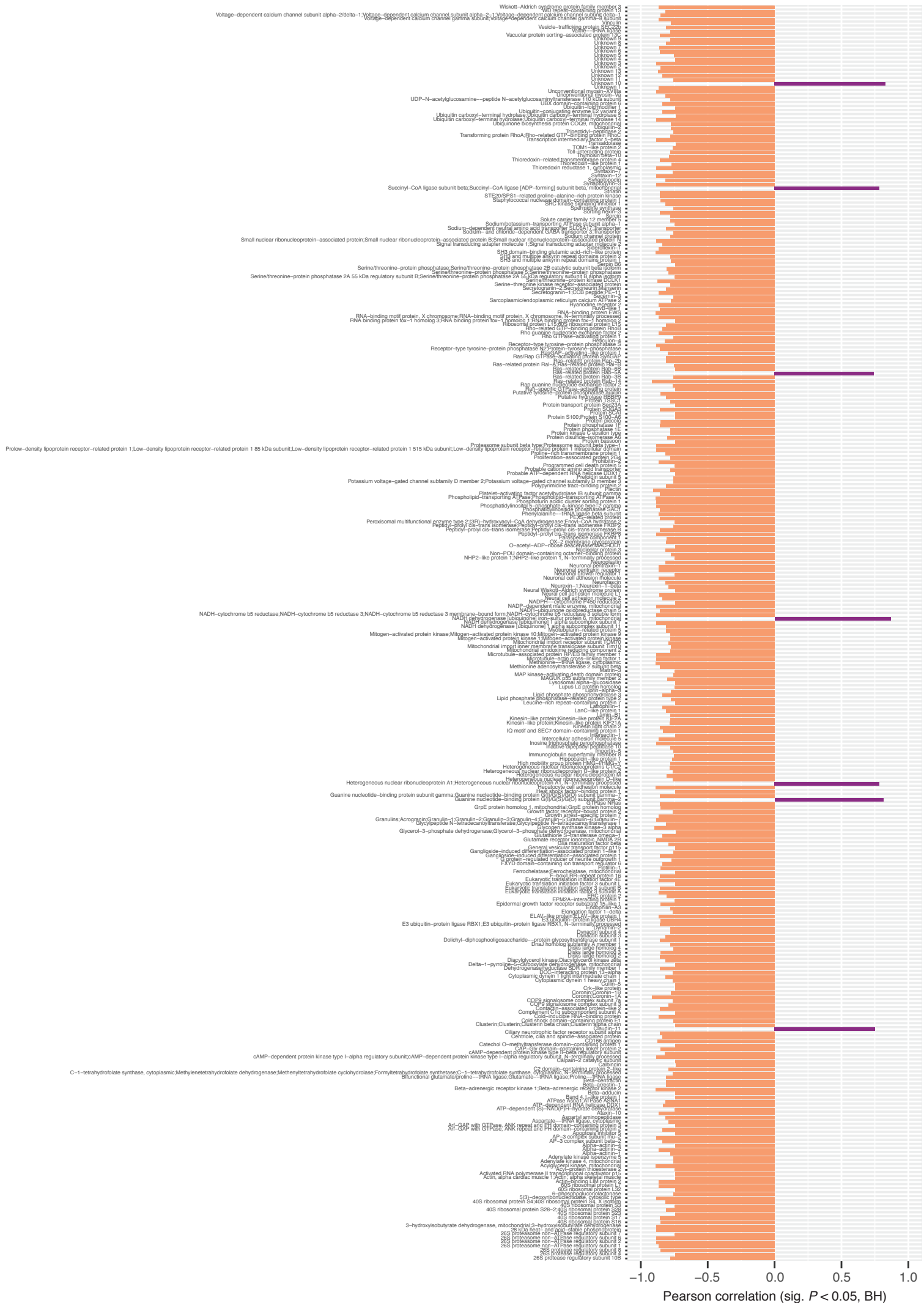

Supplement: Supplementary file 10 — Additional file 9. Metabolome-proteome correlation (Pearson). Results for Arabinose . [file 40168_2020_914_MOESM9_ESM.pdf]

## Fatty acids C6-onwards

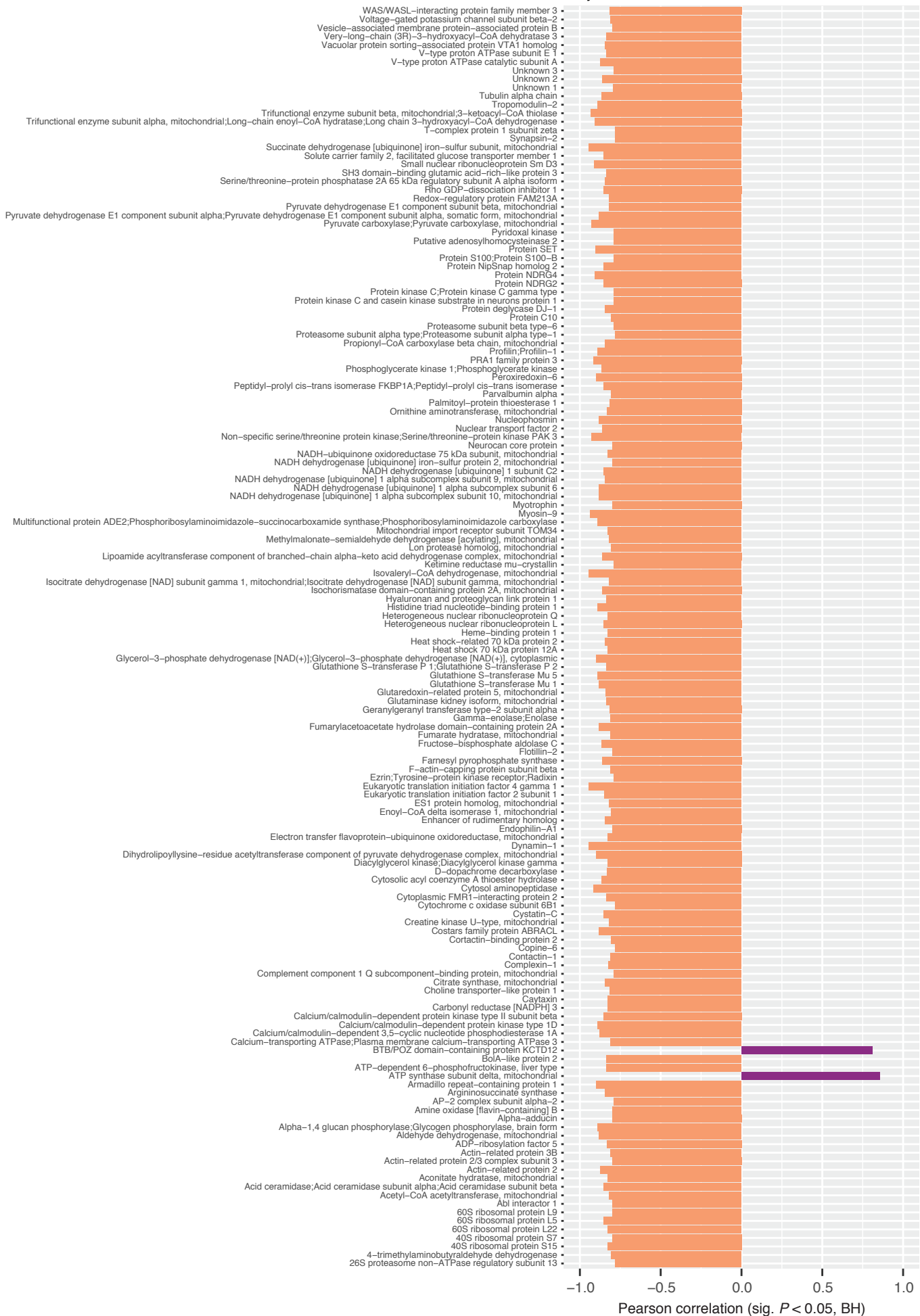

Pearson correlation (sig.  $P < 0.05$ , BH)

Supplement: Supplementary file 11 — Additional file 10. Metabolome-proteome correlation (Pearson). Results for Fatty acids C6 onwards . [file 40168_2020_914_MOESM10_ESM.pdf]

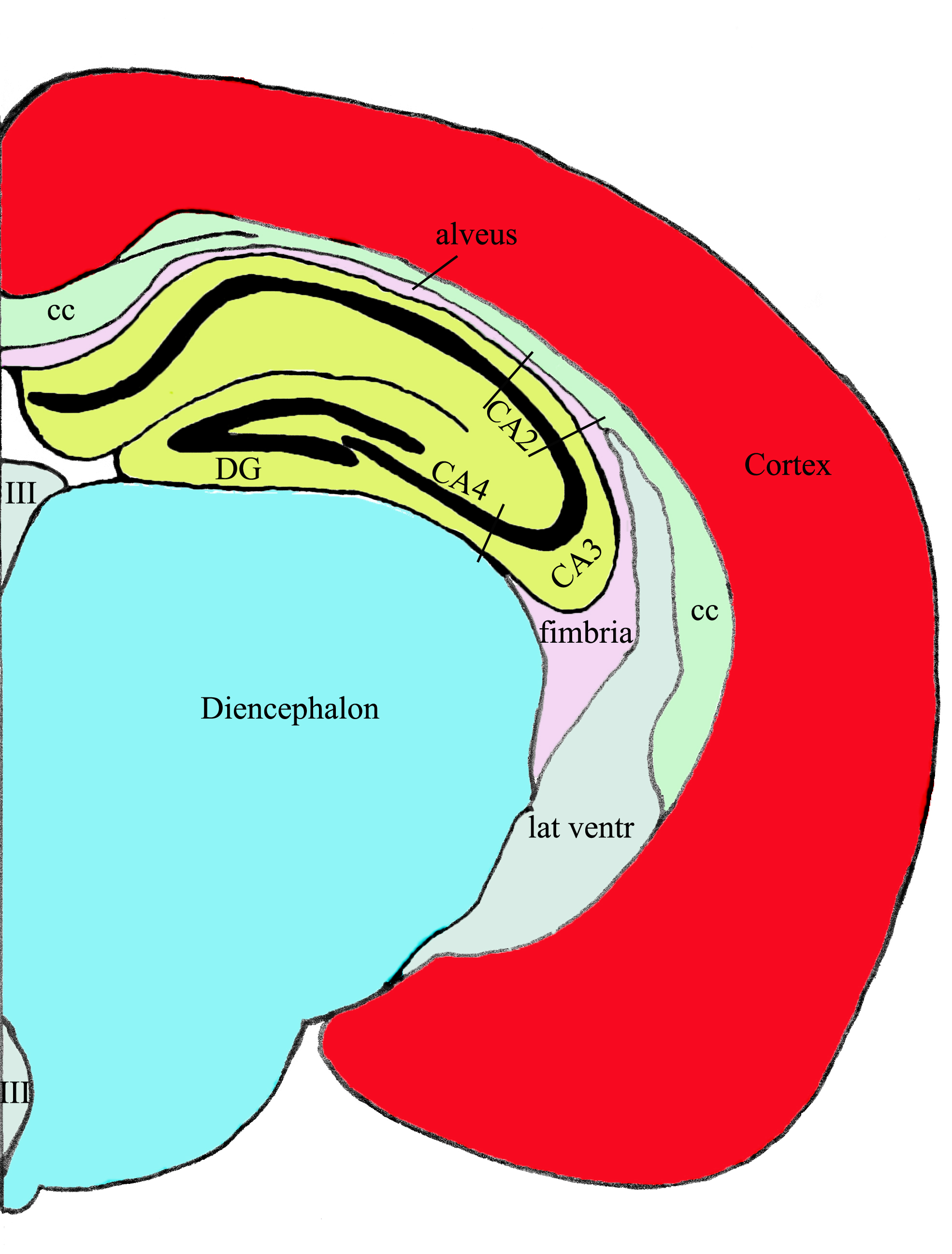

Supplement: Supplementary file 14 — Additional file 13. (Schematic of the regions of the hippocampus). Schematic frontal section of a mouse hemibrain to show the regions were confocal images were acquired to measure the GFAP and F4/80 fluorescence intensities. Images were taken from the dentate gyrus (DG), and from the CA4 and CA3 regions of the hippocampal gyrus (GFAP immunofluorescence) at -1.9 mm from the bregma. Images of the fimbria (F4/80 immunofluorescence) were acquired at -1.4 mm from the bregma. Lat ventr = lateral ventricule; cc = corpus callosum. [file 40168_2020_914_MOESM13_ESM.jpg]

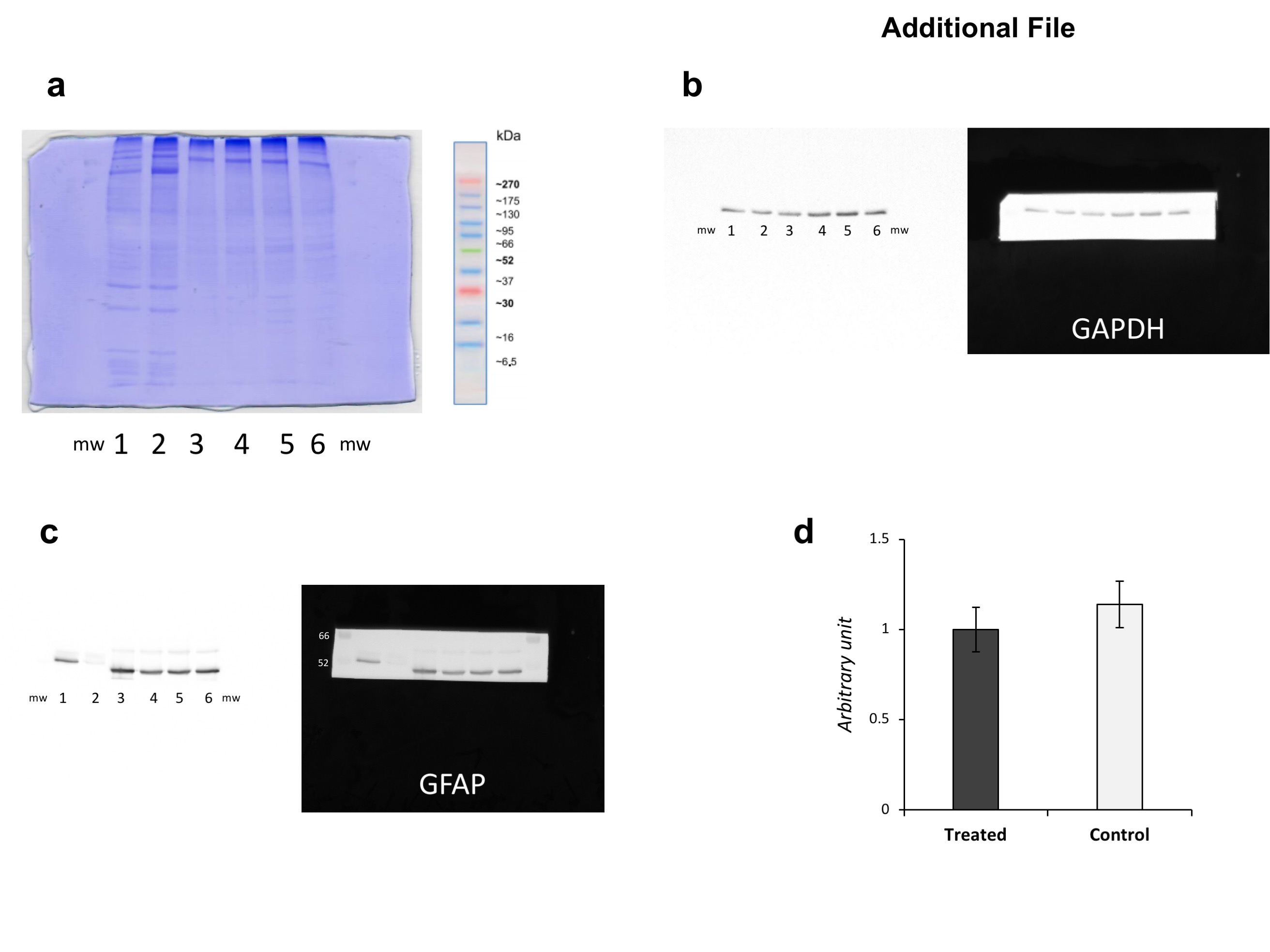

Supplement: Supplementary file 15 — Additional file 14. GFAP western blotting. Polyacrylamide (10%) gel stained with blue Coomassie with a representative image of molecular weight marker with relevant kDa (a). GAPDH visualized bands and merged with nitrocellulose membrane (b). GFAP visualized bands and merged with nitrocellulose membrane (c). In (d) a representative histogram shows levels of analysed protein both in FMT-Y and FMT-A treated and control animals. Lane 1 (positive control, DITNC1 astrocyte-derived cell line); lane 2 (negative control, BV-2 microglial cell line); lane 3 (aged mouse hippocampal proteins); lane 4 (adult mouse hippocampal protein); lane 5 (MT-aged hippocampal proteins); lane 6 (MT-adult hippocampal proteins). GFAP protein was detected approximately at 55 kDa (right blots). GAPDH (37 kDa) was used as housekeeping (left panel). Molecular weight (mw) used was SHARPMASS VII. [file 40168_2020_914_MOESM14_ESM.jpg]

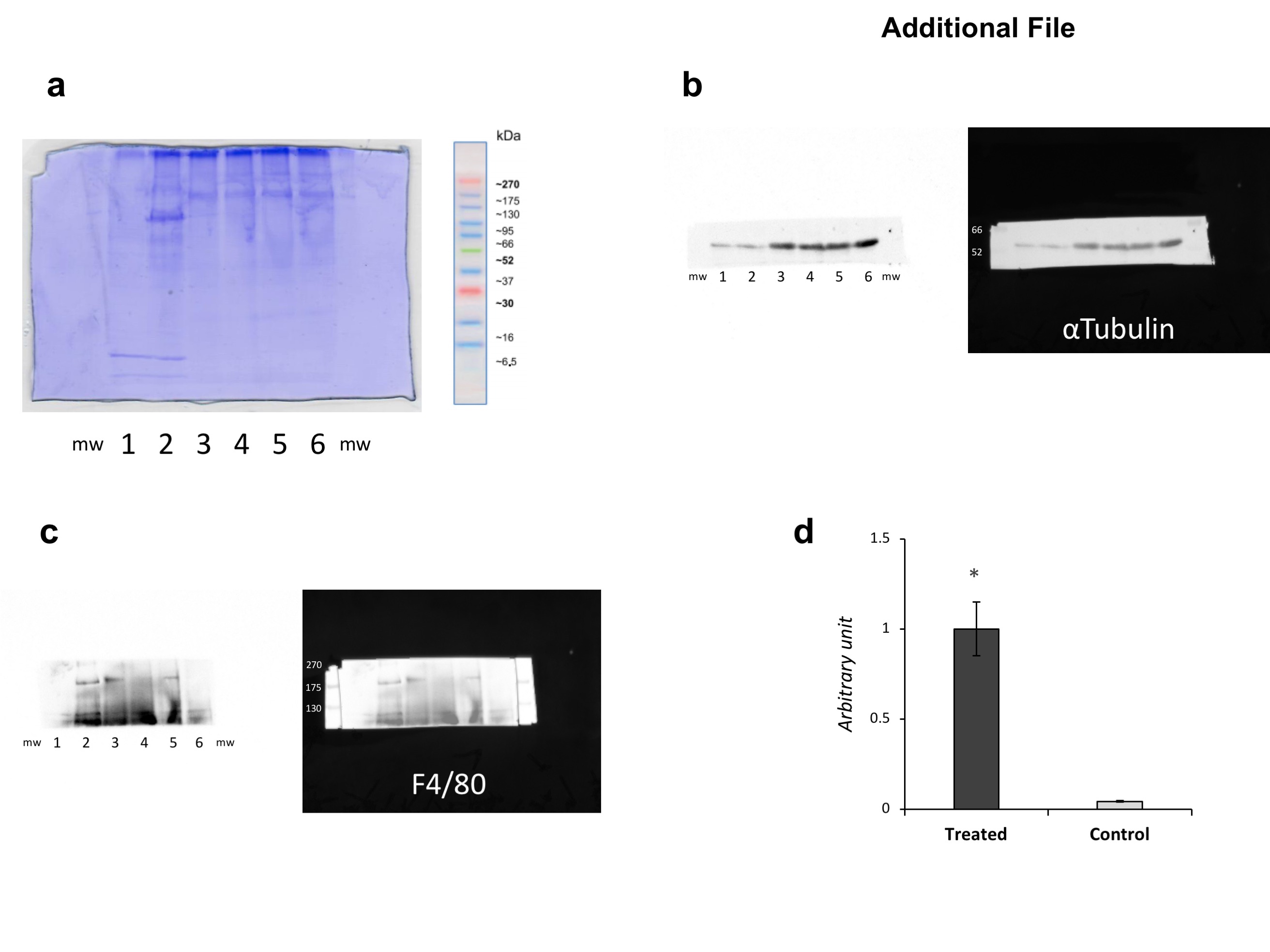

Supplement: Supplementary file 16 — Additional file 15. F4/80 western blotting. Polyacrylamide (8%) gel stained with blue Coomassie with a representative image of molecular weight marker with relevant kDa (a). GAPDH visualized bands and merged with nitrocellulose membrane (b). F4/80 visualized bands and merged with nitrocellulose membrane (c). In (d) a representative histogram shows levels of analysed protein both in FMT-Y and FMT-A treated and control animals. Lane 1 (Negative control, RBE4 brain endothelial cell line); lane 2 (positive control, BV-2 microglial cell line); lane 3 (aged mouse hippocampal proteins); lane 4 (adult mouse hippocampal protein); lane 5 (MT-aged hippocampal proteins); lane 6 (MT-adult hippocampal proteins). F4/80 protein was detected approximately at 160 kDa (right blots). αTubulin (52 kDa) was used as housekeeping (left panel). Molecular weight (mw) used was SHARPMASS VII. [file 40168_2020_914_MOESM15_ESM.jpg]

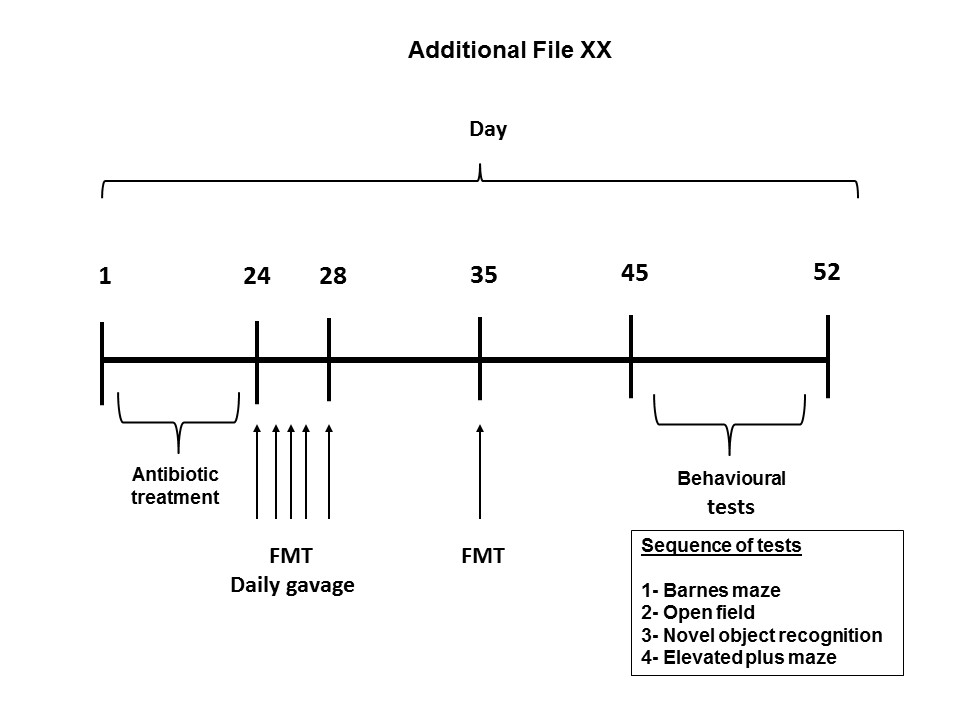

Supplement: Supplementary file 17 — Additional file 16. Summary of FMT procedure. [file 40168_2020_914_MOESM16_ESM.jpg]
